# 24-Hour Movement Behaviours (Physical Activity, Sedentary Behaviour and Sleep) Association with Glycaemic Control and Psychosocial Outcomes in Adolescents with Type 1 Diabetes: A Systematic Review of Quantitative and Qualitative Studies

**DOI:** 10.3390/ijerph20054363

**Published:** 2023-02-28

**Authors:** Mhairi Patience, Xanne Janssen, Alison Kirk, Stephanie McCrory, Eilidh Russell, William Hodgson, Megan Crawford

**Affiliations:** 1 Psychology Group, Faculty of Humanities & Social Sciences, School of Psychological Sciences & Health, University of Strathclyde, Glasgow G1 1XP, UK; 2 Physical Activity for Health Group, Faculty of Humanities & Social Sciences, School of Psychological Sciences & Health, University of Strathclyde, Glasgow G1 1XP, UK

**Keywords:** 24-hour movement behaviours, physical activity, sedentary behaviour, sleep, type 1 diabetes, adolescents, glycaemic control, quality of life, review

## Abstract

Type 1 Diabetes (T1D) is a condition requiring 24-hour management. The way in which an individual combines their 24-hour movement behaviours (24-h MBs), which is comprised of physical activity (PA), sedentary behaviour (SB), and sleep, throughout the day can have a significant impact on physical and mental health. This mixed methods systematic review aimed to investigate 24-h MBs’ relationship with glycaemic control and psychosocial outcomes in adolescents (11–18 years) with T1D. Ten databases were searched for quantitative and qualitative English language articles reporting at least one of the behaviours and their relationship with outcomes. There were no restrictions on article publication dates or study design. Articles were subjected to title and abstract screening, full text screening, data extraction and quality assessment. Data were summarised narratively, and a meta-analysis was conducted where possible. From 9922 studies, 84 were included for data extraction (quantitative (*n* = 76), qualitative (*n* = 8)). Meta-analyses revealed a significant favourable association between PA and HbA1c (−0.22 [95% CI: −0.35, −0.08; I^2^ = 92.7%; *p* = 0.001). SB had an insignificant unfavourable association with HbA1c (0.12 [95% CI: −0.06, 0.28; I^2^ = 86.1%; *p* = 0.07]) and sleep had an insignificant favourable association (−0.03 [95% CI: −0.21, 0.15; I^2^ = 65.9%; *p* = 0.34]). Importantly, no study investigated how combinations of behaviours collectively interacted and impacted on outcomes.

## 1. Introduction

Type 1 Diabetes (T1D) is a condition characterised by insulin deficiency, resulting in blood glucose levels having to be monitored and managed over the full 24-hour day using complex patterns of insulin administration. Everyday activities can affect blood glucose levels and management decisions are constantly required to maintain stable glycemia [1,2]. Although T1D can develop at any age, it most commonly develops during childhood and adolescence and is the most common form of diabetes for these age groups. The global estimate for children and adolescents (<20 years old) diagnosed with the condition is 1,211,900 with an estimated 149,500 diagnosed each year [3].

Adolescence is recognised as a particularly challenging time for individuals with T1D due to increased ownership of their condition and navigation of the usual changes occurring throughout adolescence (e.g., changes to the social environment, exploration of different lifestyles, biological changes, academic/work pressures etc.) [4]. The deterioration of metabolic control in T1D commonly occurs during adolescence with 13–25-year-olds experiencing the worst average plasma glucose concentration (HbA1c compared to other age groups) [5]. A range of mental health issues can also occur during this period, including depression and anxiety, impacting individuals’ psychosocial functioning [6,7].

Previous systematic reviews and research examining 24-hour movement behaviours (physical activity (PA), sedentary behaviour (SB), and sleep) and their relation to physical and mental health outcomes in adolescents with T1D have investigated these behaviours in isolation, with greatest emphasis placed on PA [8]. However, there has been a recent paradigm shift in the movement science literature suggesting individual movement behaviours for health should no longer be examined in isolation [9]. Instead, an integrated approach should be adopted, where all movement behaviours within the 24-hour day exist as a continuum from no movement (e.g., sleep) through low movement (e.g., sedentary behaviour) to high movement (e.g., vigorous PA (VPA)). These behaviours are considered relative or time-dependent, which means a decrease in one behaviour will result in a change in one of the other behaviours. Importantly, the way in which an individual combines these movement behaviours throughout the day can have a significant impact on physical and mental health [10]. 

In addition to the lack of systematic reviews investigating movement behaviours in youth with T1D in relation to the whole 24-hour day, there is a lack of systematic reviews examining quantitative and qualitative movement behaviour data together and their impact on T1D mental and physical health outcomes in adolescents. This would provide a comprehensive synthesis of the evidence beyond what is currently offered by a single method review by bringing together the findings of effectiveness (quantitative evidence) and experience (qualitative evidence) to enhance their usefulness to decision makers. Therefore, the aim of this study is to conduct a systematic review of quantitative and qualitative studies to comprehensively investigate 24-hour movement behaviours (individual and/or combined) and their impact on primary (glycated haemoglobin (HbA1c) and continuous glucose monitoring (CGM) metrics and quality of life (QoL)), and secondary outcomes (depressive symptoms, anxiety, stress/distress, self-management, coping, diabetes self-efficacy, family functioning, social competence) in adolescents with T1D. 

## 2. Methods

### 2.1. Scientific Rigor

The protocol for this systematic review was registered on the 24 March 2021 in PROSPERO, the international prospective register of systematic reviews (CRD42021232460). The Preferred Reporting Items for Systematic Reviews and Meta-Analyses (PRISMA) statement was used within the planning, conducting, and reporting of this review [11].

### 2.2. Inclusion Criteria

The inclusion criteria for the quantitative and qualitative studies (Table 1) were informed by PICOS (Population, Intervention, Comparison Group, Outcome, Study Design) and SPIDER (Sample, Phenomena of Interest, Design, Evaluation, Research Type) eligibility criteria, respectively [12,13]. 

### 2.3. Population/Sample

Adolescents aged 11–18 years with investigator-defined T1D of all genders, all ethnicities and all diabetes durations were included. For the qualitative studies, the primary caregivers/parents of the adolescents with diagnosed T1D were also included providing their perspectives related specifically to the adolescent. 

### 2.4. Intervention/Exposure and Phenomenon of Interest

Any individual or combined 24-hour movement behaviours (PA, SB and sleep) were included for this systematic review. Studies were included provided they were investigating habitual/usual PA. All subcategories of SB were considered (e.g., overall sedentary time, screen time and non-screen time behaviours). For sleep, all dimensions related to sleep health were included (e.g., duration, continuity or efficiency, timing, alertness/sleepiness and satisfaction/quality) [14] (Supplementary Materials Glossary of Terms S1). All outcomes of movement behaviours and related subcategories (e.g., cardiorespiratory, metabolic, muscular, morphological or motor physical fitness) were excluded. 

### 2.5. Comparisons and Design

For the quantitative studies, all control/comparison groups were included in this review provided they were within the identified population age bracket. For the qualitative studies, all qualitative methods were considered. Only data collected in habitual settings were gathered (e.g., sleep measured within a sleep lab and physical activity measured during an exercise test within a lab were excluded unless habitual movement behaviours were also reported).

### 2.6. Outcomes and Evaluation

The primary outcomes of interest for the studies were glycaemic control measured by HbA1c and/or standardised CGM metrics and QoL [15,16]. Tests/measures of QoL, Health Related Quality of Life (HRQoL) and Diabetes Specific Quality of Life providing global/total scores were reported. The secondary outcomes of interest were psychosocial (individual and family level) responses [17]. Psychosocial responses were defined as depressive symptoms, anxiety, stress/distress, self-management, coping, self-efficacy, family functioning and social competence [18] (Supplementary Materials Glossary of Terms S1). For the qualitative studies, a range of phenomena were under investigation (for example, views, behaviours, opinions, attitudes, perceptions, experiences and beliefs).

### 2.7. Study and Research Type

This systematic review collected quantitative, qualitative, and mixed method studies. Mixed method studies were only considered if data from the quantitative or qualitative components could be clearly extracted. Specifically, quantitative included experimental/interventional studies (e.g., randomised, and non-randomised) and any non-experimental/observational studies (e.g., cross-sectional/prevalence, cohort/longitudinal, case control/case reference). Additionally, all qualitative studies were included (e.g., ethnography, phenomenology, grounded theory, narrative inquiry, case studies and visual and participatory methodologies). All commentaries, reviews, editorials, meta-analysis, and diagnostic studies were excluded from this review. 

### 2.8. Search Strategy

An electronic literature search was conducted on the 7th of May 2021 in the following electronic databases: MEDLINE (Ovid), EMBASE (Ovid), Web of Science (Core Collection), APAPsychINFO (EBSCOhost), SPORTDiscus (EBSCOhost), Applied Social Sciences Index and Abstracts (ProQuest), Sports Medicine and Education Index (ProQuest) Wiley Cochrane Library, OpenGrey and Open Dissertations (EBSCOhost). All published studies up until the search date were included provided they met the inclusion criteria. For each database, a search strategy consisting of keywords and synonyms was developed using the PICO and SPIDER frameworks (Supplementary Materials Search Strategy S2).

### 2.9. Screening Process

All identified citations (*n* = 9922) were initially compiled into EndNote reference management software (https://endnote.com (accessed on 15 May 2021)) to remove duplicates [19]. The remaining references (*n* = 6513) were exported to Covidence systematic review software (https://www.covidence.org (accessed on 15 May 2021)). Further duplicates identified by Covidence were removed [*n* = 14], resulting in a total of 6499 references for title and abstract screening.

Independent screening of the studies occurred at the title and abstract stage and the full text stage by four reviewers (M.P., S.M., E.R., W.H.). Authors were contacted for missing texts. The eligibility of each study was confirmed through completion of quantitative and qualitative inclusion/exclusion checklist. Any disagreements surrounding the exclusion or inclusion of studies at each stage were discussed until a unified decision was made. If a consensus could not be reached, a third reviewer was consulted (X.J.). Cohen’s Kappa [κ] was calculated between each reviewer pair for the title and abstract (0.60–0.85) and full text screening stages (0.62–0.69) [20] (Supplementary Materials Table S1).

### 2.10. Data Extraction

The PICO (quantitative) and SPIDER (qualitative) criteria were used to inform data extraction. Sample characteristics (e.g., age, diabetes duration, HbA1c and sample size), study design (e.g., experimental, cross sectional, cohort and case-control), exposure/intervention description (e.g., movement behaviour and measurement method), primary and secondary outcomes and results data were extracted from studies (Supplementary Materials Table S2). Four reviewers completed independent data extraction for the quantitative studies (M.P., S.M., E.R., W.H.) and two reviewers completed independent extraction for the qualitative studies (M.P., S.M.). Any disagreements were discussed, and a third reviewer was consulted when a consensus could not be reached (X.J.).

### 2.11. Quality Assessment

The Critical Appraisal Skills Programme checklists were utilised to assess the methodological quality of the cohort, case-control, randomised control trials and qualitative studies [21]. The Strengthening of Reporting of Observational Studies in Epidemiology checklist was utilised for the included cross-sectional studies [22]. Assessment of each included study was performed by M.P. No studies were excluded based on quality assessment (Supplementary Materials Table S3).

### 2.12. Data Synthesis

#### 2.12.1. Narrative Synthesis of Quantitative Studies

The Excel database utilised for quantitative data extraction was initially examined by movement behaviour and respective movement behaviour constructs (Supplementary Materials Table S2). Each movement behaviour was examined in relation to the primary and secondary outcomes of interest for favourable results, unfavourable results, or results with no significance. Results were deemed favourable if the outcomes improved or indicated a trend for improvement due to higher levels of the movement behaviour. For example, for correlational results, PA’s negative correlation with HbA1c would be deemed favourable as higher PA reduced (improved) HbA1c. Additionally, for studies examining differences, results would be deemed favourable if groups with higher PA had lower (improved) HbA1c (Supplementary Materials Tables S4–S7).

#### 2.12.2. Narrative Synthesis of Qualitative Studies

Findings/themes from each qualitative study were gathered and accompanied by illustrations (e.g., quote). Findings were then grouped by common movement behaviour categories. Textual pooling was not possible due to a low number of included qualitative studies that were also very heterogenous and were therefore presented in narrative form (Supplementary Materials Table S8).

#### 2.12.3. Meta-Analysis Synthesis

Meta-analyses of quantitative studies were performed using the Meta-Essentials tool [https://www.erim.eur.nl/research-support/meta-essentials/ (accessed on 7 March 2022)]. The tool consists of a set of workbooks designed for Microsoft Excel that, based on the input, automatically produces all the required statistics, tables, figures, and more [23]. All the included studies for the meta-analysis included cross sectional associations between exposure and outcome which were derived from cross-sectional and longitudinal study designs only. For the longitudinal studies, the correlation statistic that was closest to the exposure was taken to highlight acute opposed to chronic effects of the exposure. Authors viewed this as more in keeping with associations present in cross-sectional studies. Correlation coefficients were extracted from studies and beta (b) coefficients were extracted and converted to correlation coefficients [24]. The I^2^ statistic was utilised to determine heterogeneity between studies and subsequent random-effect or fixed effect analysis (0–40% = low heterogeneity; 75–100% = significant heterogeneity) [25].

Meta-analyses were possible between the following exposure-outcome associations and their subgroups (if applicable):

- PA and HbA1c (Subgroups = Light-PA (LPA), Moderate-PA (MPA), Vigorous-PA (VPA), Moderate-Vigorous-PA (MVPA) and Total-PA (TPA)).
- SB and HbA1c (Subgroups = Computer, TV, Total Screen Time, Schoolwork and Total Sedentary Time).
- Sleep and HbA1c (Duration dimension only due to study heterogeneity).

Unfavourable associations and favourable associations were determined based on the direction of association. For PA exposures, a negative association was deemed favourable (e.g., more PA associated with lower HbA1c) and a positive association was deemed unfavourable (e.g., more PA associated with higher HbA1c). For SB exposures, a positive association was deemed favourable (e.g., less screen time associated with lower HbA1c) and a negative association was deemed unfavourable (e.g., less screen time associated with higher HbA1c). For sleep duration, a negative association was deemed favourable (e.g., increased sleep duration was associated with lower HbA1c) and a positive association was deemed unfavourable (e.g., increased sleep duration was associated with higher HbA1c). 

## 3. Results

### 3.1. Characteristics of Identified Studies

**Figure 1:**
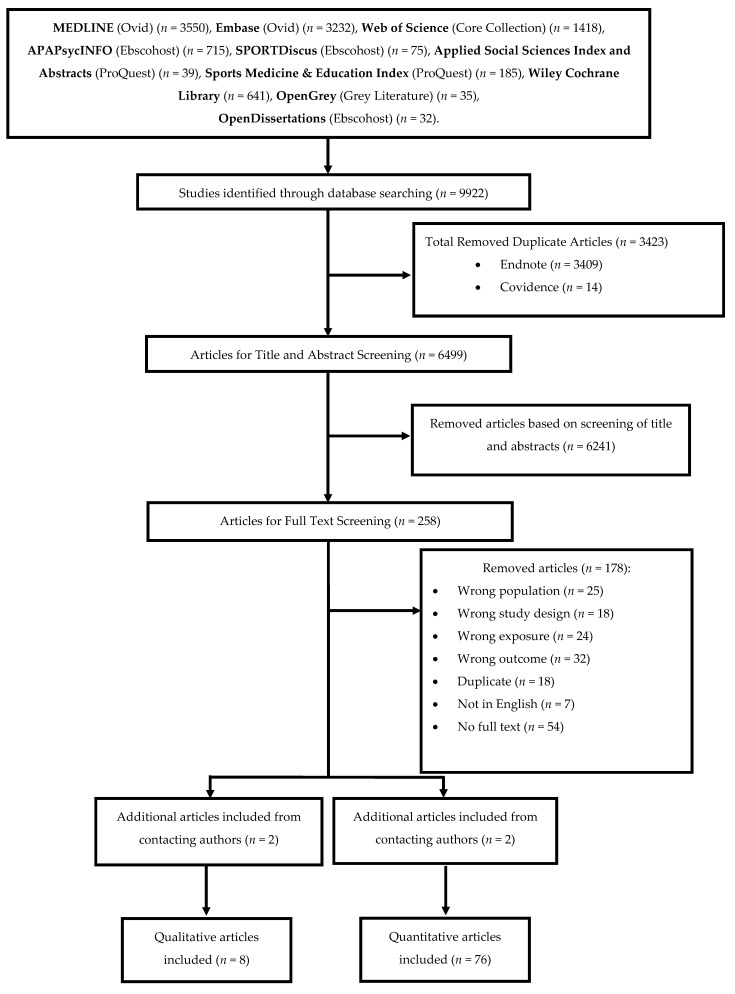
Flow chart of included studies.

In total, 9922 articles were identified from the initial search with 84 articles included for data extraction after title, abstract and full text screening (quantitative (*n* = 76) [26,27,28,29,30,31,32,33,34,35,36,37,38,39,40,41,42,43,44,45,46,47,48,49,50,51,52,53,54,55,56,57,58,59,60,61,62,63,64,65,66,67,68,69,70,71,72,73,74,75,76,77,78,79,80,81,82,83,84,85,86,87,88,89,90,91,92,93,94,95,96,97,98,99,100,101] qualitative (*n* = 8) [102,103,104,105,106,107,108,109] (Figure 1). Included quantitative articles were published between 1990–2021 [26,53] and conducted in the USA (*n* = 28) [30,33,40,41,42,44,45,46,47,51,53,54,60,69,70,77,78,81,82,83,85,86,87,92,94,97], Europe (*n* = 25) [31,32,39,43,48,49,50,56,57,58,59,61,62,63,64,67,68,75,80,89,90,91,93,96,98] UK (*n* = 6) [34,37,38,66,72,88], South America) (*n* = 5) [35,36,71,74,84], Middle East (*n* = 4) [27,76,100,101], Canada (*n* = 3) [73,79,99], Africa (*n* = 2] [26,55], New Zealand (*n* = 1) [65] and across multiple countries (*n* = 2) [28,29]. There were two experimental [52,72], 10 case-control [26,36,39,57,61,63,65,82,97,99], 11 longitudinal [30,33,40,41,46,50,60,70,77,89,90] and 53 cross-sectional studies [27,28,29,31,32,34,35,37,38,42,43,44,45,47,48,49,51,53,54,55,56,58,59,62,64,66,67,68,69,71,73,74,75,76,78,79,80,81,83,84,85,86,87,88,91,92,93,94,95,96,98,100,101]. The sample of the quantitative studies included a total of 68,203 adolescents ranging from 10–23,251 [30,49] participants per study. The mean age of participants was 14.1 years (11.1–17.6 years) [54,66], with a mean diabetes duration of 5.8 years (0.5–12.5 years) and a mean HbA1c of 8.8% (73 mmol/mol) (7.2–10.2%) [47,50]. Included qualitative articles were published between 2009–2018 [102,103,105,108]. Six analysed data thematically [102,103,106,107,108,109], one used latent content analysis [105] and one used interpretive phenomenological analysis [104]. These studies were conducted in the UK (*n* = 5) [104,106,107,108,109], USA (*n* = 3) [102,103] and Europe (*n* = 1) [105]. The total adolescent sample of the qualitative studies was 105, ranging from 11–29 participants per study [105]. These adolescents had a mean age of 12.7 years (10.8–15.56 years) [102,109], a mean diabetes duration of 5.1 years [3.8–6.2 years] [102,106] and a mean HbA1c of 8.6% [8.3–8.9%] [102,103,104]. The total sample of parents was 92, ranging from 11–25 participants per study [102,108].

### 3.2. Movement Behaviour Composition

**Figure 2:**
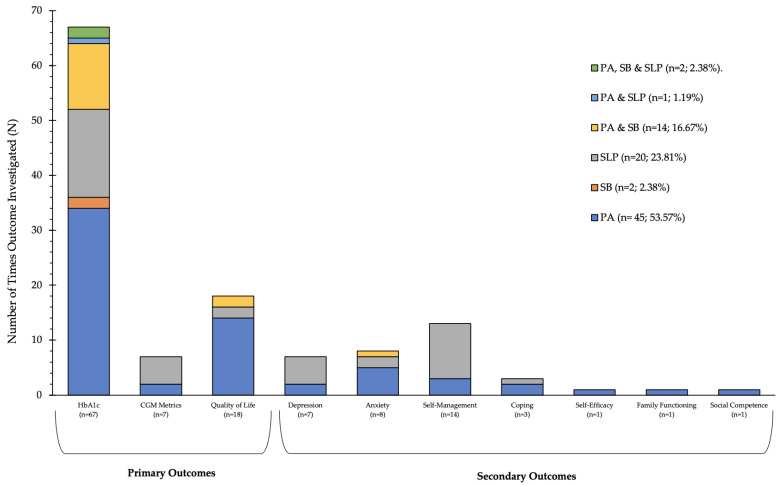
Movement Behaviours and the Addressed Primary and Secondary Outcomes of Included Studies. PA, physical activity; SB, sedentary behaviour; SLP, sleep; HbA1c, haemoglobin A1c; CGM, continuous glucose monitoring.

Half of all the included quantitative and qualitative studies were investigating PA individually (*n* = 45; 53.57%) [27,29,30,31,32,34,35,37,38,40,41,48,49,53,55,56,57,59,61,62,63,64,68,74,76,77,78,80,84,86,87,88,89,91,92,93,94,96,104,105,106,107,108,109], 2.38% (*n* = 2) [60,67] of studies were investigating SB individually and 23.81% (*n* = 20) of studies were investigating sleep individually [26,42,44,45,47,51,52,65,69,81,82,83,85,95,97,98,99,101,102,103]. A total of 16.67% (*n* = 14) of studies were investigating both PA and SB [28,33,39,43,50,54,58,66,70,72,73,75,90,100], 1.19% (*n* = 1) [46] of studies were investigating both PA and sleep and 2.38% (*n* = 2) of studies were investigating all three behaviours [36,71] (Figure 2). 

Specific physical activity constructs addressed in the included studies were TPA, MVPA, VPA, MPA and LPA. Sedentary behaviour constructs included total sedentary behaviour (SED), screen time (computer), screen time (television), sedentary behaviour (schoolwork) and screen time (TV and computer). Finally, sleep constructs addressed included duration, continuity/efficiency, timing, quality and alertness/sleepiness (Tables S4–S7).

### 3.3. Included Articles Primary and Secondary Outcome Composition

Examination of the primary outcomes showed HbA1c was addressed most frequently (*n* = 67), primarily by PA studies (*n* = 34) [27,30,31,32,34,37,38,40,41,48,49,53,55,56,61,63,64,68,74,76,78,79,80,86,88,89,92,94,96,104,106,107,108,109] followed by sleep (*n* = 16) [26,42,45,47,51,52,65,69,81,82,83,85,98,99,101,102] and then PA and SB studies (*n* = 12) [28,33,39,43,50,54,58,66,70,73,90,100]. QoL was the second most addressed outcome (*n* = 18), primarily by PA studies (*n* = 14) [29,30,35,41,55,62,63,64,76,77,93,104,106,107] followed by sleep (*n* = 2) [85,97] and PA and SB (*n* = 2) [72,75]. CGM metrics were the least frequently addressed primary outcome (*n* = 7), primarily addressed by sleep studies (*n* = 5) [44,47,65,82,85] followed by PA studies (*n* = 2) [84,91]. Examination of the secondary outcomes showed self-management was addressed most frequently (*n* = 13) [105], primarily by sleep studies (*n* = 10) [42,45,47,51,52,69,81,85,95,102]. Anxiety was the second most addressed (*n* = 8] [30,50,57,76,85,86,87,103] followed by depression (*n* = 7) [45,47,76,82,85,94,101]. The outcomes least frequently addressed were coping (*n* = 3) [85,93,109], self-efficacy (*n* = 1) [37], family functioning (*n* = 1) [106] and social competence (*n* = 1) [30] (Figure 2).

### 3.4. Physical Activity

**Figure 3:**
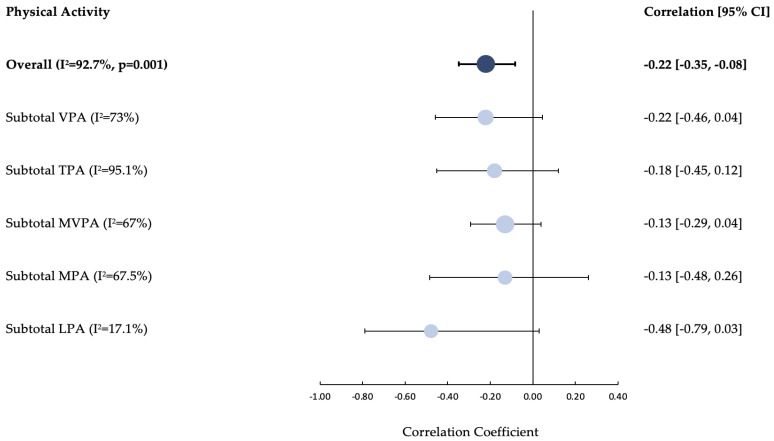
Forest Plot of the correlations between Physical Activity and Glycated Haemoglobin [HbA1c]. CI, confidence interval; LPA, light physical activity; MPA, moderate physical activity; MVPA, moderate-vigorous physical activity; TPA, total physical activity; VPA, vigorous physical activity; I^2^,statistic of heterogeneity.

Overall, PA’s impact on HbA1c was favourable in 19/60 (32%) associations and unfavourable in 2/6 (3%), most results investigating PA and HbA1c were not significant (39/60) (60%). PA’s impact on QoL was favourable in 11/15 (73%) associations, the remainder were not significant (20%). Overall, PA had no associations with depression, self-efficacy, family functioning and social competence. A mix of favourable and unfavourable associations with anxiety and self-management were observed with no clear pattern emerging (Tables S4–S7). The random effects meta-analysis to quantify associations between PA and HbA1c highlighted a significant negative association (overall pooled correlation coefficient = −0.22 [95% CI: −0.35, −0.08; I^2^ = 92.7%, *p* = 0.001]. The subgroup analysis revealed negative associations with HbA1c for each PA construct (LPA = −0.48 [95% CI: −0.79, 0.03; I^2^ = 17%]; MPA = −0.13 [95% CI: −0.48, 0.26; I^2^ = 67.5%]; MVPA = −0.13 [95% CI: −0.29, 0.04; I^2^ = 67%]; TPA = −0.18 [95% CI: −0.45, 0.12; I^2^ = 95.1%] and VPA −0.22 [95% CI: −0.46, 0.04; I^2^ = 73%]) (Figure 3). Please see Figure S1 for a forest plot of individual studies.

### 3.5. Sedentary Behaviour

The overall impact SB had on HbA1c highlighted 12/25 (48%) unfavourable associations. The only favourable associations were present for the screen time (schoolwork) construct (3/3; 100%). There were no associations between SB and QoL or any of the secondary outcomes (Tables S4–S7).

**Figure 4:**
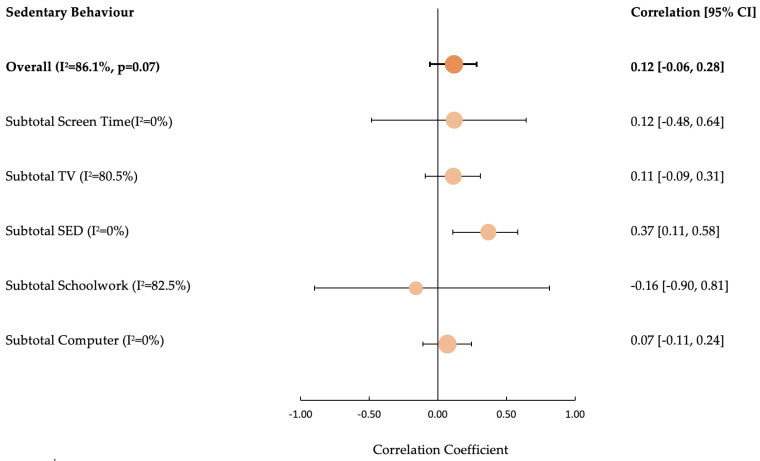
Forest Plot of the Effect of Sedentary Behaviour on Glycated Haemoglobin [HbA1c]. SED, sedentary time; CI, confidence interval; I^2^, statistic of heterogeneity.

The random effects meta-analysis to quantify associations between SB and HbA1c highlighted an insignificant positive association (overall pooled correlation coefficient = 0.12 [95% CI: −0.06, 0.28; I^2^ = 86%; *p* = 0.07]). Subgroup analysis of SB constructs revealed positive association for screen time computer (0.07 [95% CI: −0.11, 0.24; I^2^ = 0%]), total sedentary time (0.37 [95% CI: 0.11, 0.58; I^2^ = 0%]), screen time television (0.11 [95% CI: −0.09, 0.31; I^2^ = 80.5%]) and total screen time (0.12 [95% CI: −0.48, 0.64; I^2^ = 0%]). However, a negative association was observed between screen time schoolwork and HbA1c (−0.16 [95% CI: −0.90, 0.81; I^2^ = 82.5%]) (Figure 4). Please see Figure S2 for a forest plot of individual studies.

### 3.6. Sleep

**Figure 5:**
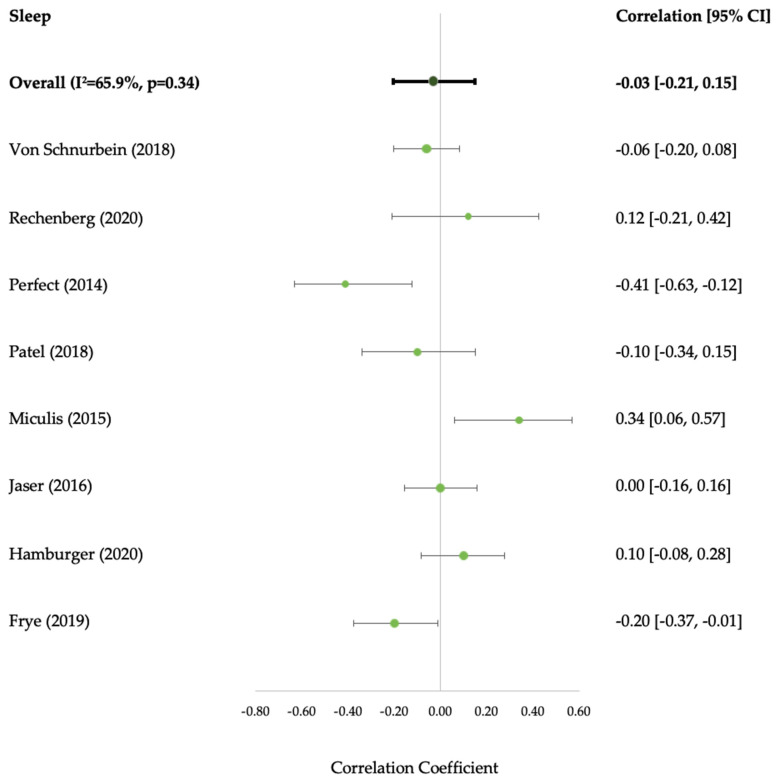
Forest Plot of the Effect of Sleep Duration on Glycated Haemoglobin [HbA1c]. CI, confidence interval; I^2^**,** statistic of heterogeneity [42,45,51,71,81,83,85,98].

Sleep’s overall impact on HbA1c highlighted 5/32 (16%) favourable and 3/32 (9%) unfavourable associations; the majority were not associated (75%). Sleep’s impact on QoL was favourable in 0/2 associations. There was a mix of favourable (5/19) (26%) and unfavourable (3/19) (16%) associations with self-management and favourable associations with depression (5/8) (63%). No associations were observed with anxiety or coping (Tables S4–S7). It was only possible to examine sleep duration construct in the meta-analysis, and this association was not significant (−0.03 [95% CI: −0.21, 0.15; I^2^ = 65.9%; *p* = 0.34]) (Figure 5).

### 3.7. Qualitative Studies

PA was investigated most frequently (*n* = 6; 75%) [104,105,106,107,108,109] followed by sleep (*n* = 2; 25%) [102,103] with no studies focusing primarily on SB (Supplementary Materials Table S8). Adolescents with T1D and their parents discussed the positive impact PA had in relation to management of glycaemic control in 4/5 studies [104,106,108,109]. This was highlighted by both adolescents with T1D and their parents/caregivers:

‘‘It helps to sort of control. I don’t really know why but I felt that um, if I’m doing more exercise um I can normally keep my levels at a more consistent rate”.

[Adolescent, p4] [104]

“What we’ve learned is that physical activity keeps the spikes and the lows more moderate so you don’t fluctuate as much… the physical activity just makes that more stable”

[Caregiver, p4] [108]

There was discussion from the adolescents with T1D surrounding PA’s positive role for improving quality of life in all studies addressing the outcome [3/3] [104,106,107]. This was expressed through the description of positive holistic feelings:

“I feel quite satisfied”, “cheerful”, “I like walking because it really relaxes me”

[Adolescents, p7] [107]

“When you’re doing exercise you know you’re helping your body as well as yourself”

[Adolescent, p5] [104]

There was also a glimpse of discussion from parents surrounding PA role in supporting adolescents to cope and how the behaviour might strengthen family functioning [106,109]:

“a way of getting out his anger”

[Caregiver, p6] [109]

“because it’s funner with other people, like you can keep motivated, but you can also have a laugh while you’re doing it…’,

[Adolescent, p153] [106]

Both adolescents and their caregivers expressed fluctuations in glycaemic control as a barrier to obtaining sleep and the anxieties of extreme glucose fluctuations while sleeping [102,103]:

“Usually [TEEN] sleeps fine, unless his blood sugar’s high. Then he’s up every couple hours”.

[Caregiver p123] [102]

“I panic a lot because one night my blood sugar dropped in the middle of the night and I ended up like having a seizure”.

[Adolescent, p550] [103]

“My mother sets an alarm for 2:30 in the morning and she comes and checks me so she doesn’t want me to have to get up in the middle of the night”.

[Adolescent, p550] [103]

Finally, sleep was also discussed in relation to self-management behaviours by adolescents and caregivers [102]. They discuss how curtailments to sleep result in increased lethargy which has subsequent impact on management behaviours (e.g., blood glucose checks). Interestingly, one adolescent highlighted the impact reduced sleep has on their sitting levels:

“I’m not my usual self. I don’t like do things, I just kinda sit”.

[Adolescent, p546] [102]

“the lack of sleep would probably cause [her teen] to not be able to keep track of his blood sugars or test when he needs—it probably causes him to be a little lazy about it”

[Caregiver, p546] [102]

## 4. Discussions

To our knowledge, this is the first systematic review to examine the full spectrum of 24-hour movement behaviours in relation to glycaemic control and psychosocial outcomes in adolescents with T1D. We found no studies investigating how combinations of behaviours collectively interact and impact on any of the primary or secondary outcomes.

The results of our study confirmed previous research demonstrating isolated movement behaviours are related to HbA1c and psychosocial outcomes. Both the quantitative and qualitative findings reported higher levels of PA improved glycaemic control and quality of life [110,111,112,113]. Additionally, we identified from the qualitative findings the potential benefit of PA on family functioning that was not otherwise highlighted in the quantitative findings. Higher levels of SB worsened HbA1c in most studies, aside from screen time for schoolwork activities which indicated a trend for improved HbA1c. This could be explained by the conscientiousness personality trait associated with homework completion in adolescents which has been found to be related to positive T1D self-management behaviours and thus glycaemic control [114]. The findings from this systematic review highlighted a potential decrease in HbA1c with increased levels of sleep duration; however, the meta-analysis was insignificant and this aligns with previous research [115]. The inclusion of the qualitative studies allowed for a deeper insight into this bi-directional relationship with participants discussing experiences of glycaemic control issues that resulted in interruptions to their sleep. The mixed findings surrounding the direct association of sleep duration and HbA1c might be explained by the mediating effects of self-management activities [116]. Improvements in sleep might improve self-management activities resulting in the subsequent improvement of glycaemic control. Self-management was the most frequently investigated secondary outcome, largely in relation to sleep. We found in quantitative findings both long and short sleep duration, poor sleep quality and delayed timing (e.g., later bedtime) worsened self-management activities. This aligned with our qualitative findings where curtailments of sleep were believed to decrease self-management behaviours.

Although 20.4% of studies reported on two or more movement behaviours, these were examined separately from one another in relation to our outcomes. Most studies investigating more than one behaviour focused on examining both PA and SB. Only 2.4% of the included studies focused solely on SB, a theme consistent with findings from other systematic reviews [111]. The lack of SB focus is also present in current T1D management guidance, illustrated in the International Society for Pediatric and Adolescent Diabetes (ISPAD) PA guidelines. ISPAD recommends youth participate in mostly MVPA and VPA for 60 min/day; however, there are no specific SB guidelines. Instead, minimal SB guidance is made available within the same guidelines, simply stating: “*sedentary lifestyle behaviors should be routinely screened for and discouraged in the diabetic clinic*” [8]. While the research and guidance may suggest SB is deemed less important than PA, optimism lies in the fact these behaviours are often investigated in unison, indicating appreciation for the close alignment of these behaviours. This holds promise for the adoptability of a 24-hour approach by researchers and key stakeholders. Additionally, the qualitative results of this study also indicate adolescents might be willing to adopt a 24-hour approach with one adolescent recognising the interdependency of behaviours (increased levels of sitting after disruptions to sleep).

Research is beginning to investigate the levels of 24-hour movement behaviours relative to one another, rather than as individual entities in populations with chronic conditions [117]. However, no studies to our knowledge have investigated how the behaviours interact and subsequently impact outcomes in adolescents with chronic conditions, including type 1 diabetes. Evidence outside this population consistently illustrates PA, SB and sleep are linked, they impact one another, and this has consequences for physical and mental health [10]. 24-hour movement behaviour guidelines were first published in 2016 with a specific focus on children and youth [118]. These guidelines acknowledge the small percentage MVPA accounts for within the 24 h day (<5%), *move* away from the previous dominant focus on MVPA and additionally consider the percentage of sleep (40%), SB (40%) and LPA (15%) [119]. Adopting a 24-hour approach for adolescents with T1D is a logical step in the management of the condition for several reasons. A 24-hour approach would allow PA to remain one of the cornerstones of T1D management while also placing equal and required emphasis on the benefits of reducing SB. Additionally, although there are mixed findings surrounding the exact relationship sleep may have with glycaemic control, sleep behaviour and diabetes research is gaining traction. This is highlighted by the recent calls for T1D sleep guidance and the widely accepted difficulties both caregivers and adolescents with T1D have with sleep [65,120]. Finally, and importantly, sleep is a necessary behaviour not only to adolescents with T1D but all populations. Other T1D management tools are often prioritised over PA (e.g., diet and medication) despite the behaviour’s recognised benefits. Utilising a 24-hour approach where each behaviour is weighted and known to interact would promote the necessity and importance of PA and SB through sleep association. Essentially, PA could ‘piggyback’ on the necessity of sleep, resulting in higher prioritisation of PA and the associated improvements in outcomes.

### Strengths and Limitations

This systematic review provided a comprehensive exploration of the full spectrum of movement behaviours in relation to a range of primary and secondary outcomes, creating novel and holistic findings. Throughout the review process, Covidence meta-analysis software was utilised that is designed to facilitate organisation of studies which enhanced the rigorous assessments of each stage and the reliability of this systematic review’s findings. A mixed methods approach was utilised, allowing for in depth examination of effectiveness from the quantitative studies and real-life experience from the qualitative studies. This allowed gaps between effectiveness and experience to be highlighted which exploited possible areas for future research (e.g., PA association with family functioning; the direction of relationship between sleep and glycaemic control) strengthens current findings (e.g., higher PA improves glycaemic control and quality of life) and ultimately enhances usefulness of findings to decision makers.

While the comprehensiveness of this review allowed for a holistic view of associations, there were a range of different terminologies that overlapped, creating difficulty in collating the findings. Studies examining movement behaviours should recognise the multidimensional nature of each behaviour, ensure they are reporting on the exact construct of interest and report this in a manner consistent with previous research or overarching gold standard guidance (e.g., Sedentary Behaviour Research Network or American Psychological Association Thesaurus). This uniformity of reporting would increase the overall strength of findings. While this review incorporated a range of sleep health dimensions, it did not incorporate the recent addition of the regularity dimension, and future research might investigate this dimension in relation to our outcomes. It would have been beneficial to also examine additional outcomes (e.g., hypoglycaemia, hyperglycaemia, risk factors for co-morbidities) to add a further perspective to these results. However, incorporating these outcomes in a review looking at a range of different behaviours was not feasible due to time and resource constraints.

It is important to acknowledge the limitation of HbA1c as an outcome measure. HbA1c only provides an average level of blood glucose over the past 2–3 months and little information can be ascertained regarding the frequency, duration or amplitude of intra-day (within-day) and inter-day (between-day) glycaemic excursions [16]. CGMs have increased in availability and accuracy over the last century and have facilitated diabetic glycaemic control. They produce real-time measurements of the glucose level excursions HbA1c overlooks, allowing for immediate action to address raised or lowered glucose levels that can subsequently prevent potential acute incidents (e.g., hypoglycaemia and hyperglycaemia). However, in this systematic review minimal studies investigated the movement behaviours’ impact on glycaemic control assessed via CGM metrics despite the increased availability of the device and the recommended standardised metrics that aim to guide clinicians, patients and researchers in using, analysing, and reporting CGM data.

## 5. Conclusions

This systematic review highlighted that no studies to date have investigated how combinations of behaviours collectively interact and impact on any of the primary or secondary outcomes in adolescents with T1D. Monitoring the full spectrum of 24-hour movement behaviours would allow for a comprehensive understanding of how the cumulation and weighting of each behaviour might interact and impact on important outcomes for adolescents with T1D. Future research should investigate the association between 24-hour movement behaviours (measured via accelerometer), glycaemic control and psychosocial outcomes. Additionally, measuring glucose control via both HbA1c and CGM glucose metrics would aid in the comprehensive investigation by providing a detailed objective and continuous pattern of glucose over the full 24-hour period. Finally, qualitative studies investigating the knowledge, awareness and feasibility of a 24-hour movement behaviour approach would aid in understanding how adolescents with T1D might adopt this type of approach.

## Figures and Tables

**Table 1:** Eligibility criteria for the quantitative and qualitative studies.

PICOS Statement (Quantitative)	SPIDER Statement (Qualitative)
Population: Adolescents (11–18 years) with researcher defined diagnosed type 1 diabetes	Sample: Adolescents (11–18 years) with researcher defined diagnosed type 1 diabetes and primary caregivers/parents of adolescents with researcher defined diagnosed type 1 diabetes
Intervention/Exposure: Individual or combined 24-hour movement behaviours	Phenomenon of Interest: At least one 24-hour movement behaviour theme
Comparisons: All control/comparison groups	Design: All qualitative methods
Outcomes: HbA1c, CGM metrics and QoL	Evaluation: Beliefs, experiences, attitudes, behaviours and interactions etc.
Study: Interventional/experimental and observational	Research Type: Qualitative

HbA1c, haemoglobin A1c; CGM, continuous glucose monitor; QoL, quality of life.

## Data Availability

Not applicable.

## Supplementary Materials

The following supporting information can be downloaded at: https://www.mdpi.com/article/10.3390/ijerph20054363/s1, Glossary of Terms S1; Search Strategy S2; Table S1: Inter-Rater reliability between reviewers; Table S2: Characteristics of Quantitative Studies; Table S3: Quality Assessment; Tables S4–S7: Movement Behaviours Associations with Primary and Secondary Outcomes; Table S8: Characteristics of Qualitative Studies; Figure S1: Forest Plot of Physical Activity Individual Studies; Figure S2: Forest Plot of Sedentary Behaviour Individual Studies.
