# Supplementary material for: 24-Hour Movement Behaviours (Physical Activity, Sedentary Behaviour and Sleep) Association with Glycaemic Control and Psychosocial Outcomes in Adolescents with Type 1 Diabetes: A Systematic Review of Quantitative and Qualitative Studies
Source: Int J Environ Res Public Health. 2023 Feb 28;20(5):4363. doi: 10.3390/ijerph20054363 (PMC10001999; doi:10.3390/ijerph20054363)

**Figure S2:** Forest Plot of Sedentary Behaviour Individual Studies. CI, confidence interval; $I^2$, statistic of heterogeneity

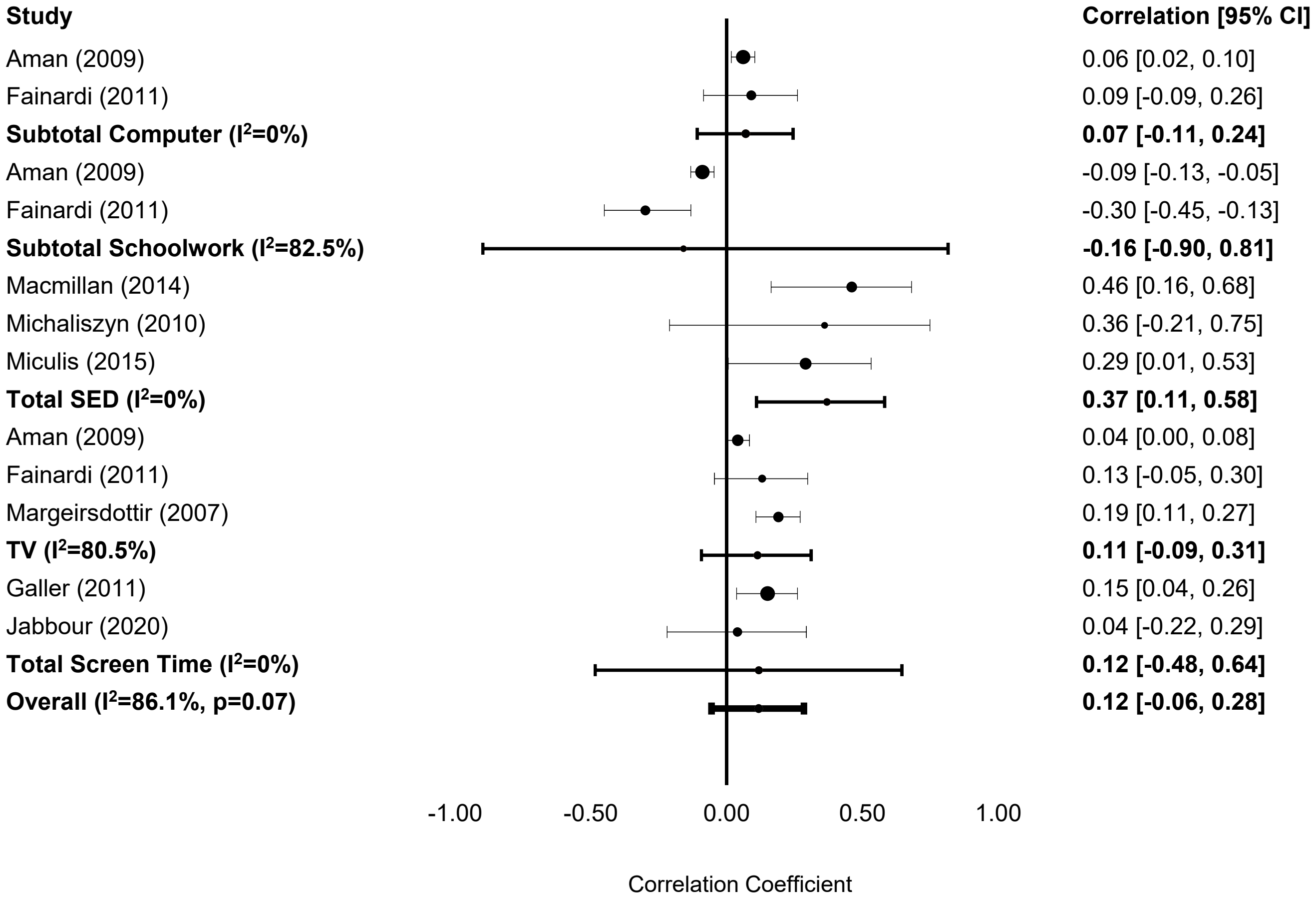

Supplement: Supplementary file 1 [file ijerph-20-04363-s001.zip › Figure S2- Forest Plot of Sedentary Behaviour Individual Studies copy.pdf]
