# Supplementary material for: 24-Hour Movement Behaviours (Physical Activity, Sedentary Behaviour and Sleep) Association with Glycaemic Control and Psychosocial Outcomes in Adolescents with Type 1 Diabetes: A Systematic Review of Quantitative and Qualitative Studies
Source: Int J Environ Res Public Health. 2023 Feb 28;20(5):4363. doi: 10.3390/ijerph20054363 (PMC10001999; doi:10.3390/ijerph20054363)
Supplement: Supplementary file 1 [file ijerph-20-04363-s001.zip › Glossary of Terms S1.pdf]

Supplementary Material 1
Glossary of Terms

Glossary of Sleep Health Terms

Sleep duration: The total amount of sleep obtained per 24 hours

Sleep continuity or efficiency: The ease of falling asleep and returning to sleep

Timing: The placement of sleep within the 24-hour day

Alertness/sleepiness: The ability to maintain attentive wakefulness

Satisfaction/Quality: The subjective assessment of "good" or "poor" sleep

Glossary of Primary Outcomes

HbA1c - measures the average level of blood glucose over the past 2-3 months to indicate long-term glycaemic control

CGM Metrics - standardised metrics and targets to guide clinicians, patients and researchers in using, analysing, and reporting CGM data to comprehensively assess glycaemic control

1) Number of days CGM worn
2) Percentage of time CGM is active
3) Mean glucose
4) Estimated A1C
5) Glycaemic variability (%Coefficient Variation or Standard Deviation)
6) Time Above Range (Level 2)
7) Time Above Range (Level 1)
8) Time In Range
9) Time Below Range
10) Time Below Range
11) Low Blood Glucose Indicator and High Blood Glucose Indicator (risk indices)
12) Episodes (hypoglycaemia and hyperglycaemia)
13) Area under the curve
14) Time blocks (24-h, day, night)

Quality of Life - the extent to which a person obtains satisfaction from life. The following are important for a good quality of life: emotional, material, and physical well-being; engagement in interpersonal relations; opportunities for personal (e.g., skill) development; exercising rights and making self-determining lifestyle choices; and participation in society[3]:

1) *Health-Related Quality of Life* - an individual's or a group's perceived physical and mental health over time.
2) *Diabetic Quality of Life* – health related quality of life specific to diabetic population.

Glossary of Secondary Outcomes

Depression - a negative affective state, ranging from unhappiness and discontent to an extreme feeling of sadness, pessimism, and despondency, that interferes with daily life.

Anxiety - an emotion characterized by apprehension and somatic symptoms of tension in which an individual anticipates impending danger, catastrophe, or misfortune

Stress - the physiological or psychological response to internal or external stressors. Stress involves changes affecting nearly every system of the body, influencing how people feel and behave.

Distress - the negative stress response, often involving negative affect and physiological reactivity: a type of stress that results from being overwhelmed by demands, losses, or perceived threats.

Self-Management - an individual's control of his or her behaviour, particularly regarding the pursuit of a specific objective (e.g., weight loss).

Social Competence - effectiveness or skill in interpersonal relations and social situations, increasingly considered an important component of mental health. Social competence involves the ability to evaluate social situations and determine what is expected or required; to recognize the feelings and intentions of others; and to select social behaviours that are most appropriate for that given context.

Coping - the use of cognitive and behavioural strategies to manage the demands of a situation when these are appraised as taxing or exceeding one's resources or to reduce the negative emotions and conflict caused by stress.

Family Functioning - the social and structural properties of the global family environment, specifically family interactions/relationships (e.g., family conflict, cohesion, adaptability, organisation and quality of communication)
