# Supplementary material for: 24-Hour Movement Behaviours (Physical Activity, Sedentary Behaviour and Sleep) Association with Glycaemic Control and Psychosocial Outcomes in Adolescents with Type 1 Diabetes: A Systematic Review of Quantitative and Qualitative Studies
Source: Int J Environ Res Public Health. 2023 Feb 28;20(5):4363. doi: 10.3390/ijerph20054363 (PMC10001999; doi:10.3390/ijerph20054363)
Supplement: Supplementary file 1 [file ijerph-20-04363-s001.zip › Search Strategy S2.pdf]

Supplementary Material 2: Search Strategies

- An electronic literature search was conducted on **07/05/2021** in the following electronic databases: MEDLINE (Ovid), EMBASE (Ovid), Web of Science (Core Collection), APAPsycINFO (EBSCOhost), SPORTDiscus (EBSCOhost), Applied Social Sciences Index and Abstracts (ProQuest), Sports Medicine & Education Index (ProQuest) Wiley Cochrane Library, OpenGrey and Open Dissertations (EBSCOhost).
- For each database, a search strategy consisting of keywords and synonyms was developed using the PICO (Population, Intervention/Exposure, Comparison and Outcome) and SPIDER (Sample, Phenomenon of Interest, Design, Evaluation and Research type) frameworks.
- Some of the outcomes searched for this review were very specific and not available as a subject term or related narrower term in databases-controlled vocabulary (e.g. the continuous glucose monitor metrics of interest). Therefore, all developed keywords and synonyms were included in each search and combined using the "AND" and "OR" boolean operators. Additionally, the use of "wildcards" were used to enhance the searches.
- Then to ensure complete comprehensiveness of the search, identified keywords for this systematic review (**Table 1**) were searched for in the controlled vocabulary of each database and specific database functions/tools were utilised, if available. For example, in MEDLINE and EMBASE (Ovid Platform), the key term "Type 1 Diabetes" was "mapped" to match terms with the databases-controlled vocabulary. This returned the subject headings "Diabetes Mellitus, Type 1" and "insulin dependent diabetes mellitus," respectively. These terms were then "exploded" (exp) to retrieve results containing the subject heading in combination with all of its narrower, more specific subheadings.
- A summary of the keywords and their combinations used for each database search is highlighted in **Table 1**.

Table 1

Summary of Keywords and Combinations Used for each Search

1. Type 1 Diabetes
2. Adolescent
3. Physical Activity
4. Sedentary Behaviour
5. Sleep
6. 24-hour Movement Behaviour
7. Health Behaviour or Health Knowledge or Health Attitude or Health Practice (*Broader Variety of Phrases to Comprehensively Incorporate both Qualitative and Quantitative Studies*)
8. HbA1c, CGM Metrics and Quality of Life

9. Depressive Symptoms, Anxiety, Stress, Self-Management, Coping, Self-Efficacy, Family Functioning and Social Competence
10. 1 and 2 (*Population/Sample*)
11. 3 or 4 or 5 or 6 or 7 (*Intervention/Exposure/Phenomenon of Interest*)
12. 8 or 9 (*Primary and Secondary Outcomes*)
13. 10 and 11 and 12 (*Results*)

Web of Science (1,418 results) – Core Collection

Notes:

- Topic (TS) search field selected

#1 TS=(“Type 1 Diabetes” OR “insulin-dependent diabetes” OR T1DM OR IDDM OR “type 1” OR “type I” OR “juvenile onset diabetes” OR “child onset diabetes”)
#2 TS=(adolescent* OR youth* OR juvenile* OR teen* OR “young adult*” OR “young people” OR minor* OR pubescent* OR “emerging adult*”)
#3 TS=(“physical* activ*” OR exercis* OR “moderate intensity” OR “light intensity” OR “vigorous intensity” OR “very vigorous intensity” OR “high intensity” OR workout OR “physical training” OR sport* OR “strength training” OR “strength activit*” OR “resistance training” OR “resistance activit*” OR “aerobic training” OR “aerobic activit*” OR “anaerobic training” OR “anaerobic activit*” OR “flexibility training” OR “flexibility activit*” OR “endurance training” OR “endurance activit*” OR “physical education” OR PE OR walking)
#4 TS=(“sedentary behav*” OR sit* OR “screen time” OR sedentary)
#5 TS=(sleep* OR alertness OR wakefulness)
#6 TS=("24-hour movement behav*" OR "integrated behav*")
#7 TS=(“health behav*” OR “health knowledge” OR “health attitude*” OR “health practice*”)
#8 TS=(HbA1c OR A1c OR “glyc* h$emoglobin” OR glycoh$emoglobin OR “glyc$emic control” OR “glucose control” OR “h$emoglobin A1c” OR “mean glucose” OR “mean daily glucose” OR hypoglyc$emia OR hyperglyc$emia OR “time in hypoglyc$emic range” OR “time in level 1 hypoglyc$emic range” OR “time in level 2 hypoglyc$emic range” OR “time below range” OR TBR OR “time in target range” OR “time in range” OR TIR OR “time in hyperglyc$emic range” OR “time in level 1 hyperglyc$emic range” OR “time in level 2 hyperglyc$emic range” OR TAR OR “glucose variability” OR “glyc$emic variability” OR “estimated A1c” OR eA1C OR “glucose management indicator” OR GMI OR “hypoglyc$emic episodes” OR “hyperglyc$emic episodes” OR “area under the curve” OR AUC OR “risk of hypoglyc$emia” OR LBGI OR “risk of hyperglyc$emia” OR HBGI OR “time blocks” OR “quality of life” OR QoL OR well-being)
#9 TS=(depress* OR anxiet* OR stress* OR distress* OR self-management OR self-care OR self-regulations OR self-monitoring OR *adherence OR *compliance OR cope OR coping OR resilien* OR self-efficacy OR “behavioural control” OR confidence OR self-esteem OR “family functioning” OR

"family conflict" OR "family cohesion" OR "family flexibility" OR "family relation*" OR "family communication" OR "family problem solving" OR "family support" OR "family responsibility" OR "parental involvement" OR "family environment" OR "family adaptability" OR "social competence" OR "social skills" OR "social development" OR "social communication")
#9 #1 and #2
#10 #3 or #4 or #5 or #6 or #7
#11 #8 or #9
#12 #10 and #11 and #12

MEDLINE (3,550 results) – Ovid Platform

Notes:

- Keyword search was selected
- Abstract (.ab) search field selected
- "Mapping" and "Exploding" to *all* subheadings were applied for keywords of interest available in the database (**Table 1**)

1 (Type 1 Diabetes or insulin dependent diabetes or T1DM or IDDM or type 1 or type I or juvenile onset diabetes or child onset diabetes).ab. or exp Diabetes Mellitus, Type 1/
2 (adolescent* or youth* or juvenile* or teen* or young adult* or young people or minor* or pubescent or emerging adult*).ab. or exp Adolescent/ or exp Young Adult/ or exp Minors/
3 (physical* activ* or exercis* or moderate intensity or light intensity or vigorous intensity or very vigorous intensity or high intensity or workout or physical training or sport* or strength training or strength activit* or resistance training or resistance activit* or aerobic training or aerobic activit* or anaerobic training or anaerobic activit* or flexibility training or flexibility activit* or endurance training or endurance activit* or physical education or PE or walking).ab. or exp Exercise/
4 (sedentary behav* or sit* or screen time or sedentary).ab or exp Sedentary Behavior/
5 (sleep* or alertness or wakefulness).ab. or exp Sleep/
6 (24-hour movement behav* or integrated behav*).ab.
7 (health behav* or health knowledge or health attitude* or health practice*).ab or exp Health Behavior/ or exp Health Knowledge, Attitudes, Practice/ or exp Attitude to Health/
8 (HbA1c or A1c or glyc* h?emoglobin or glycoh?emoglobin or glyc?emic control or glucose control or h?emoglobin A1c or mean glucose or mean daily glucose or hypoglyc?emia or hyperglyc?emia or hypoglyc?emic range or time below range or TBR or time in range or TIR or hyperglyc?emic range or TAR or glucose variability or glyc?emic variability or estimated A1c or eA1C or glucose management indicator or GMI or hypoglyc?emic episodes or hyperglyc?emic episodes or area under the curve or AUC or risk of hypoglyc?emia or LBGI or risk of hyperglyc?emia or HBGI or time blocks or quality of life or QoL or well-being).ab or exp Glycated Hemoglobin A/ or exp Quality of Life/ or exp Blood Glucose/ or exp Hypoglycemia/ or exp Hyperglycemia/

9 (depress* or anxiet* or stress* or distress* or self-management or self-care or self-regulations or self-monitoring or adherence or compliance or cope or coping or resilien* or self-efficacy or behavioural control or confidence or self-esteem or family functioning or family conflict or family cohesion or family flexibility or family relation* or family communication or family problem solving or family support or family responsibility or parental involvement or family environment or family adaptability or social competence or social skills or social development or social communication).ab or exp Depression/ or exp Anxiety/ or exp Stress, Psychological/ or exp Psychological Distress/ or exp Self-Management/ or exp Medication Adherence/ or exp Self Care/ or exp Self Efficacy/ or exp Family Relations/ or exp Social Skills/ or exp Adaptation, Psychological/
10 1 and 2
11 3 or 4 or 5 or 6 or 7
12 8 or 9
13 10 and 11 and 12

EMBASE (3,232 results) – Ovid Platform

Notes:

- Keyword search was selected
- Abstract (.ab.) search field selected
- "Mapping" and "Exploding" to *all* subheadings were applied for keywords of interest available in the database (**Table 1**)

1 (Type 1 Diabetes or insulin dependent diabetes or T1DM or IDDM or type 1 or type I or juvenile onset diabetes or child onset diabetes).ab. or exp insulin dependent diabetes mellitus/
2 (adolescent* or youth* or juvenile* or teen* or young adult* or young people or minor* or pubescent or emerging adult*).ab. or exp adolescent/
3 (physical* activ* or exercis* or moderate intensity or light intensity or vigorous intensity or very vigorous intensity or high intensity or workout or physical training or sport* or strength training or strength activit* or resistance training or resistance activit* or aerobic training or aerobic activit* or anaerobic training or anaerobic activit* or flexibility training or flexibility activit* or endurance training or endurance activit* or physical education or PE or walking).ab or exp physical activity/
4 (sedentary behav* or sit* or screen time or sedentary).ab. or exp sedentary time/ or exp sedentary lifestyle/
5 (sleep* or alertness or wakefulness).ab. or exp sleep/
6 (24-hour movement behav* or integrated behav*).ab.
7 (health behav* or health knowledge or health attitude* or health practice*).ab. or exp health behavior/ or exp attitude to health/
8 (HbA1c or A1c or glyc* h?emoglobin or glycoh?emoglobin or glyc?emic control or glucose control or h?emoglobin A1c or mean glucose or mean daily glucose or hypoglyc?emia or hyperglyc?emia or hypoglyc?emic range or time below range or TBR or time in range or TIR or hyperglyc?emic range or

TAR or glucose variability or glyc?emic variability or estimated A1c or eA1C or glucose management indicator or GMI or hypoglyc?emic episodes or hyperglyc?emic episodes or area under the curve or AUC or risk of hypoglyc?emia or LBGI or risk of hyperglyc?emia or HBGI or time blocks or quality of life or QoL or well-being).ab. or exp hemoglobin A1c/ or exp quality of life/ or exp glucose blood level/
9 (depress* or anxiet* or stress* or distress* or self-management or self-care or self-regulations or self-monitoring or adherence or compliance or cope or coping or resilien* or self-efficacy or behavioural control or confidence or self-esteem or family functioning or family conflict or family cohesion or family flexibility or family relation* or family communication or family problem solving or family support or family responsibility or parental involvement or family environment or family adaptability or social competence or social skills or social development or social communication).ab. or exp depression/ or exp anxiety/ or exp stress/ or exp distress syndrome/ or exp patient compliance/ or exp self concept/ or exp family relation/ or exp social competence/ or exp coping behavior/
10 1 and 2
11 3 or 4 or 5 or 6 or 7
12 8 or 9
13 10 and 11 and 12

APAPsycInfo (715 results) – EBSCOHost Platform

Notes:

- Unqualified Search conducted
- ‘Boolean/Phrase Search Mode’ selected for each search
- Apply Related Words and Apply Equivalent Subjects ‘expanders’ applied for each search
- Search Informed by

S1 (“Type 1 Diabetes” OR “insulin-dependent diabetes” OR T1DM OR IDDM OR “type 1” OR “type I” OR “juvenile onset diabetes” OR “child onset diabetes”)
S2 (adolescent* OR youth* OR juvenile* or teen* OR young adult* OR “young people” OR minor* OR pubescent OR “emerging adult*”)
S3 (“physical* activ*” OR exercis* OR “moderate intensity” OR “light intensity” OR “vigorous intensity” OR “very vigorous intensity” OR “high intensity” OR workout OR “physical training” OR sport* OR “strength training” OR “strength activit*” OR “resistance training” OR “resistance activit*” OR “aerobic training” OR “aerobic activit*” OR “anaerobic training” OR “anaerobic activit*” OR “flexibility training” OR “flexibility activit*” OR “endurance training” OR “endurance activit*” OR “physical education” OR PE OR walking)
S4 (“sedentary behav*” OR sit* OR “screen time” OR sedentary)
S5 (sleep* OR alertness OR wakefulness)
S6 (“24-hour movement behav*” OR “integrated behav*”)
S7 (“health behav*” or “health knowledge” or “health attitude*” or “health practice*”)

S8 (HbA1c OR A1c OR “glyc* h#emoglobin” OR glycoh#emoglobin OR “glyc#emic control” or “glucose control” or “h#emoglobin A1c” OR “mean glucose” OR “mean daily glucose” OR hypoglyc#emia OR hyperglyc#emia OR “time in hypoglyc#emic range” OR “time in level 1 hypoglyc#emic range” OR “time in level 2 hypoglyc#emic range” OR “time below range” OR TBR OR “time in target range” OR “time in range” OR TIR OR “time in hyperglyc#emic range” OR “time in level 1 hyperglyc#emic range” OR “time in level 2 hyperglyc#emic range” OR TAR OR “glucose variability” OR “glyc#emic variability” OR “estimated A1c” OR eA1C OR “glucose management indicator” OR GMI OR “hypoglyc#emic episodes” OR “hyperglyc#emic episodes” OR “area under the curve” OR AUC OR “risk of hypoglyc#emia” OR LBGI OR “risk of hyperglyc#emia” OR HBGI OR “time blocks” OR “quality of life” OR QoL OR well-being)
S9 (depress* OR anxiet* OR stress* OR distress* OR self-management OR self-care OR self-regulations OR self-monitoring OR adherence OR compliance OR cope OR coping OR resilien* OR self-efficacy OR “behavioural control” OR confidence OR self-esteem OR “family functioning” OR “family conflict” OR “family cohesion” OR “family flexibility” OR “family relation*” OR “family communication” OR “family problem solving” OR “family support” OR “family responsibility” OR “parental involvement” OR “family environment” OR “family adaptability” OR “social competence” OR “social skills” OR “social development” OR “social communication”)
S10 S1 AND S2
S11 S3 OR S4 OR S5 OR S6 OR S7
S12 S8 OR S9
S13 S10 AND S11 AND S12

SPORTDiscus (75 results) - EBSCOHost Platform

Notes:

- Unqualified Search conducted
- ‘Boolean/Phrase Search Mode’ selected for each search
- Apply Related Words and Apply Equivalent Subjects ‘expanders’ applied for each search

S1 (“Type 1 Diabetes” OR “insulin-dependent diabetes” OR T1DM OR IDDM OR “type 1” OR “type I” OR “juvenile onset diabetes” OR “child onset diabetes”)
S2 (adolescent* OR youth* OR juvenile* or teen* OR young adult* OR “young people” OR minor* OR pubescent OR “emerging adult*”)
S3 (“physical* activ*” OR exercis* OR “moderate intensity” OR “light intensity” OR “vigorous intensity” OR “very vigorous intensity” OR “high intensity” OR workout OR “physical training” OR sport* OR “strength training” OR “strength activit*” OR “resistance training” OR “resistance activit*” OR “aerobic training” OR “aerobic activit*” OR “anaerobic training” OR “anaerobic activit*” OR “flexibility training” OR “flexibility activit*” OR “endurance training” OR “endurance activit*” OR “physical education” OR walking OR PE)
S4 (“sedentary behav*” OR sit* OR “screen time” OR sedentary)

S5 (sleep* OR alertness OR wakefulness)
S6 (“24-hour movement behav*” OR “integrated behav*”)
S7 (“health behav*” OR “health knowledge” OR “health attitude*” OR “health practice*”)
S8 (HbA1c OR A1c OR “glyc* h#emoglobin” OR glycoh#emoglobin OR “glyc#emic control” or “glucose control” or “h#emoglobin A1c” OR “mean glucose” OR “mean daily glucose” OR hypoglyc#emia OR hyperglyc#emia OR “time in hypoglyc#emic range” OR “time in level 1 hypoglyc#emic range” OR “time in level 2 hypoglyc#emic range” OR “time below range” OR TBR OR “time in target range” OR “time in range” OR TIR OR “time in hyperglyc#emic range” OR “time in level 1 hyperglyc#emic range” OR “time in level 2 hyperglyc#emic range” OR TAR OR “glucose variability” OR “glyc#emic variability” OR “estimated A1c” OR eA1C OR “glucose management indicator” OR GMI OR “hypoglyc#emic episodes” OR “hyperglyc#emic episodes” OR “area under the curve” OR AUC OR “risk of hypoglyc#emia” OR LBGI OR “risk of hyperglyc#emia” OR HBGI OR “time blocks” OR “quality of life” OR QoL OR well-being)
S9 (depress* OR anxiet* OR stress* OR distress* OR self-management OR self-care OR self-regulations OR self-monitoring OR adherence OR compliance OR cope OR coping OR resilien* OR self-efficacy OR “behavioural control” OR confidence OR self-esteem OR “family functioning” OR “family conflict” OR “family cohesion” OR “family flexibility” OR “family relation*” OR “family communication” OR “family problem solving” OR “family support” OR “family responsibility” OR “parental involvement” OR “family environment” OR “family adaptability” OR “social competence” OR “social skills” OR “social development” OR “social communication”)
S10 S1 AND S2
S11 S3 OR S4 OR S5 OR S6 OR S7
S12 S8 OR S9
S13 S10 AND S11 AND S12

OpenDissertations (Grey Literature; 32 results) - EBSCOHost Platform

Notes:

- Unqualified Search conducted
- ‘Boolean/Phrase Search Mode’ selected for each search
- Apply Related Words and Apply Equivalent Subjects ‘expanders’ applied for each search

S1 (“Type 1 Diabetes” OR “insulin-dependent diabetes” OR T1DM OR IDDM OR “type 1” OR “type I” OR “juvenile onset diabetes” OR “child onset diabetes”)
S2 (adolescent* OR youth* OR juvenile* or teen* OR young adult* OR “young people” OR minor* OR pubescent OR “emerging adult*”)
S3 (“physical* activ*” OR exercis* OR “moderate intensity” OR “light intensity” OR “vigorous intensity” OR “very vigorous intensity” OR “high intensity” OR workout OR “physical training” OR sport* OR “strength training” OR “strength activit*” OR “resistance training” OR “resistance activit*” OR “aerobic training” OR “aerobic activit*” OR “anaerobic training” OR “anaerobic activit*” OR “flexibility training”

OR “flexibility activit*” OR “endurance training” OR “endurance activit*” OR “physical education” OR walking OR PE)
S4 (“sedentary behav*” OR sit* OR “screen time” OR sedentary)
S5 (sleep* OR alertness OR wakefulness)
S6 (“24-hour movement behav*” OR “integrated behav*”)
S7 (“health behav*” OR “health knowledge” OR “health attitude*” OR “health practice*”)
S8 (HbA1c OR A1c OR “glyc* h#emoglobin” OR glycoh#emoglobin OR “glyc#emic control” or “glucose control” OR “h#emoglobin A1c” OR “mean glucose” OR “mean daily glucose” OR hypoglyc#emia OR hyperglyc#emia OR “time in hypoglyc#emic range” OR “time in level 1 hypoglyc#emic range” OR “time in level 2 hypoglyc#emic range” OR “time below range” OR TBR OR “time in target range” OR “time in range” OR TIR OR “time in hyperglyc#emic range” OR “time in level 1 hyperglyc#emic range” OR “time in level 2 hyperglyc#emic range” OR TAR OR “glucose variability” OR “glyc#emic variability” OR “estimated A1c” OR eA1C OR “glucose management indicator” OR GMI OR “hypoglyc#emic episodes” OR “hyperglyc#emic episodes” OR “area under the curve” OR AUC OR “risk of hypoglyc#emia” OR LBGI OR “risk of hyperglyc#emia” OR HBGI OR “time blocks” OR “quality of life” OR QoL OR well-being)
S9 (depress* OR anxiet* OR stress* OR distress* OR self-management OR self-care OR self-regulations OR self-monitoring OR adherence OR compliance OR cope OR coping OR resilien* OR self-efficacy OR “behavioural control” OR confidence OR self-esteem OR “family functioning” OR “family conflict” OR “family cohesion” OR “family flexibility” OR “family relation*” OR “family communication” OR “family problem solving” OR “family support” OR “family responsibility” OR “parental involvement” OR “family environment” OR “family adaptability” OR “social competence” OR “social skills” OR “social development” OR “social communication”)
S10 S1 AND S2
S11 S3 OR S4 OR S5 OR S6 OR S7
S12 S8 OR S9
S13 S10 AND S11 AND S12

Applied Social Sciences Index and Abstracts (39 results)– ProQuest Platform

Notes:

- Abstract search field selected (AB)
- The database thesaurus or controlled vocabulary of subject terms was searched for key terms of interest available in the database (**Table 1**) and then ‘exploded’

S1 AB(“Type 1 Diabetes” OR “insulin dependent diabetes” OR T1DM OR IDDM OR “type 1” OR “type I” OR “juvenile onset diabetes” OR “child onset diabetes”) OR MAINSUBJECT.EXACT.EXPLODE("Insulin dependent diabetes mellitus")

S2 AB(adolescent* OR youth* OR juvenile* or teen* OR “young adult*” OR “young people” OR minor* OR pubescent OR “emerging adult*”) OR MAINSUBJECT.EXACT.EXPLODE("Adolescents") OR MAINSUBJECT.EXACT.EXPLODE("Young adults")

S3 AB(“physical* activ*” OR exercis* OR “moderate intensity” OR “light intensity” OR “vigorous intensity” OR “very vigorous intensity” OR “high intensity” OR workout OR “physical training” OR sport* OR “strength training” OR “strength activit*” OR “resistance training” OR “resistance activit*” OR “aerobic training” OR “aerobic activit*” OR “anaerobic training” OR “anaerobic activit*” OR “flexibility training” OR “flexibility activit*” OR “endurance training” OR “endurance activit*” OR “physical education” OR PE OR walking) OR MAINSUBJECT.EXACT.EXPLODE("Physical activity")

S4 AB(“sedentary behav*” OR sit* OR “screen time” OR sedentary) OR MAINSUBJECT.EXACT.EXPLODE("Sedentary people")

S5 AB(sleep* OR alertness OR wakefulness) OR MAINSUBJECT.EXACT.EXPLODE(“Sleep”)

S6 AB(“24-hour movement behav*” OR “integrated behav*”)

S7 AB(“health behav*” OR “health knowledge” OR “health attitude*” OR “health practice*”) OR MAINSUBJECT.EXACT.EXPLODE("Health behaviour") OR MAINSUBJECT.EXACT.EXPLODE("Health beliefs")

S8 AB(HbA1c OR A1c OR “glyc* h?emoglobin” OR glycoh?emoglobin OR “glyc?emic control” OR “glucose control” OR “mean glucose” OR “mean daily glucose” OR hypoglyc?emia OR hyperglyc?emia OR “time in hypoglyc?emic range” OR “time in level 1 hypoglyc?emic range” OR “time in level 2 hypoglyc?emic range” OR “time below range” OR TBR OR “time in target range” OR “time in range” OR TIR OR “time in hyperglyc?emic range” OR “time in level 1 hyperglyc?emic range” OR “time in level 2 hyperglyc?emic range” OR TAR OR “glucose variability” OR “glyc?emic variability” OR “estimated A1c” OR eA1C OR “glucose management indicator” OR GMI OR “hypoglyc?emic episodes” OR “hyperglyc?emic episodes” OR “area under the curve” OR AUC OR “risk of hypoglyc?emia” OR LBGI OR “risk of hyperglyc?emia” OR HBGI OR “time blocks” OR “quality of life” OR QoL OR well-being) OR MAINSUBJECT.EXACT.EXPLODE("Glycaemic control") OR MAINSUBJECT.EXACT.EXPLODE("Quality of life")

S9 AB(depress* OR anxiet* OR stress* OR distress* OR self-management OR self-care OR self-regulations OR self-monitoring OR adherence OR compliance OR cope OR coping OR resilien* OR self-efficacy OR “behavioural control” OR confidence OR self-esteem OR “family functioning” OR “family conflict” OR “family cohesion” OR “family flexibility” OR “family relation*” OR “family communication” OR “family problem solving” OR “family support” OR “family responsibility” OR “parental involvement” OR “family environment” OR “family adaptability” OR “social competence” OR “social skills” OR “social development” OR “social communication”) OR MAINSUBJECT.EXACT.EXPLODE("Anxiety") OR MAINSUBJECT.EXACT.EXPLODE("Depression") OR MAINSUBJECT.EXACT.EXPLODE("Psychological distress") OR MAINSUBJECT.EXACT.EXPLODE("Stress") OR MAINSUBJECT.EXACT.EXPLODE("Selfcare") OR MAINSUBJECT.EXACT.EXPLODE("Adherence") OR MAINSUBJECT.EXACT.EXPLODE("Family functioning") OR MAINSUBJECT.EXACT.EXPLODE("Family relationships") OR MAINSUBJECT.EXACT.EXPLODE("Coping") OR MAINSUBJECT.EXACT.EXPLODE("Social

competence") OR MAINSUBJECT.EXACT.EXPLODE("Social skills") OR MAINSUBJECT.EXACT.EXPLODE("Adherence")
S10 S1 AND S2
S11 S3 OR S4 OR S5 OR S6 OR S7
S12 S8 OR S9
S13 S10 AND S11 AND S12

Sports Medicine & Education Index (185 results) – ProQuest Platform

Notes:

- Abstract search field selected (AB)
- The database thesaurus or 'controlled vocabulary of subject terms' was searched for key terms of interest available in the database (**Table 1**). The exploded option was not available.

S1 AB("Type 1 Diabetes" OR "insulin dependent diabetes" OR T1DM OR IDDM OR "type 1" OR "type I" OR "juvenile onset diabetes" OR "child onset diabetes") OR MAINSUBJECT.EXACT("Diabetes")
S2 AB(adolescent* OR youth* OR juvenile* or teen* OR "young adult*" OR "young people" OR minor* OR pubescent OR "emerging adult*") OR MAINSUBJECT.EXACT("Teenagers") OR MAINSUBJECT.EXACT("Young adults")
S3 AB("physical* activ*" OR exercis* OR "moderate intensity" OR "light intensity" OR "vigorous intensity" OR "very vigorous intensity" OR "high intensity" OR workout OR "physical training" OR sport* OR "strength training" OR "strength activit*" OR "resistance training" OR "resistance activit*" OR "aerobic training" OR "aerobic activit*" OR "anaerobic training" OR "anaerobic activit*" OR "flexibility training" OR "flexibility activit*" OR "endurance training" OR "endurance activit*" OR "physical education" OR PE OR walking) OR MAINSUBJECT.EXACT("Exercise")
S4 AB("sedentary behave*" OR sit* OR "screen time" OR sedentary) OR MAINSUBJECT.EXACT("Screen time")
S5 AB(sleep* OR alertness OR wakefulness) OR MAINSUBJECT.EXACT("Sleep")
S6 AB("24-hour movement behav*" OR "integrated behav*")
S7 AB("health behav*" OR "health knowledge" OR "health attitude*" OR "health practice*") OR MAINSUBJECT.EXACT("Health behavior")
S8 AB(HbA1c OR A1c OR "glyc* h*emoglobin" OR glycoh?emoglobin OR "glyc?emic control" OR "glucose control" OR "mean glucose" OR "mean daily glucose" OR hypoglyc?emia OR hyperglyc?emia OR "time in hypoglyc?emic range" OR "time in level 1 hypoglyc?emic range" OR "time in level 2 hypoglyc?emic range" OR "time below range" OR TBR OR "time in target range" OR "time in range" OR TIR OR "time in hyperglyc?emic range" OR "time in level 1 hyperglyc?emic range" OR "time in level 2 hyperglyc?emic range" OR TAR OR "glucose variability" OR "glyc?emic variability" OR "estimated A1c" OR eA1C OR "glucose management indicator" OR GMI OR "hypoglyc?emic episodes" OR "hyperglyc?emic episodes" OR "area under the curve" OR AUC OR "risk of hypoglyc?emia" OR LBGI OR "risk of hyperglyc?emia" OR HBGI OR "time blocks" OR "quality of life"

OR QoL OR well-being) OR MAINSUBJECT.EXACT("Glucose monitoring") OR MAINSUBJECT.EXACT("Quality of life")

S9 AB(depress* OR anxiet* OR stress* OR distress* OR self-management OR self-care OR self-regulations OR self-monitoring OR adherence OR compliance OR cope OR coping OR resilien* OR self-efficacy OR “behavioural control” OR confidence OR self-esteem OR “family functioning” OR “family conflict” OR “family cohesion” OR “family flexibility” OR “family relation*” OR “family communication” OR “family problem solving” OR “family support” OR “family responsibility” OR “parental involvement” OR “family environment” OR “family adaptability” OR “social competence” OR “social skills” OR “social development” OR “social communication”) OR MAINSUBJECT.EXACT("Self-medication") OR MAINSUBJECT.EXACT("Patient compliance") OR MAINSUBJECT.EXACT("Social skills") OR MAINSUBJECT.EXACT("Anxiety") OR MAINSUBJECT.EXACT("Anxiety disorders") OR MAINSUBJECT.EXACT("Families & family life") OR MAINSUBJECT.EXACT("Mental depression") OR MAINSUBJECT.EXACT("Stress") OR MAINSUBJECT.EXACT("Coping") OR MAINSUBJECT.EXACT("Self esteem")

S10 S1 AND S2

S11 S3 OR S4 OR S5 OR S6 OR S7

S12 S8 OR S9

S13 S10 AND S11 AND S12

Wiley Cochrane Library (641 results)

Notes:

- Abstract search fields selected (:ab)
- “MeSH” headings (controlled vocabulary) were searched and “exploded” [mh]

#1 (“Type 1 Diabetes” OR “insulin-dependent diabetes” OR T1DM OR IDDM OR “type 1” OR “type I” OR “juvenile onset diabetes” OR “child onset diabetes”):ab OR [mh “Diabetes Mellitus, Type 1”]

#2 (adolescent* OR youth* OR juvenile* or teen* OR young NEXT adult* OR “young people” OR minor* OR pubescent OR emerging NEXT adult*):ab OR [mh Adolescent]

#3 (physical* NEXT activ* OR exercis* OR “moderate intensity” OR “light intensity” OR “vigorous intensity” OR “very vigorous intensity” OR “high intensity” OR workout OR “physical training” OR sport* OR “strength training” OR strength NEXT activit* OR “resistance training” OR resistance NEXT activit* OR “aerobic training” OR aerobic NEXT activit* OR “anaerobic training” OR anaerobic NEXT activit* OR “flexibility training” OR flexibility NEXT activit* OR “endurance training” OR endurance NEXT activit* OR “physical education” OR PE OR walking):ab OR [mh Exercise]

#4 (sedentary NEXT behav* OR sit* OR “screen time” OR sedentary):ab OR [mh “Sedentary Behavior”]

#5 (sleep* OR alertness OR wakefulness):ab OR [mh Sleep]

#6 (24 NEXT hour NEXT movement NEXT behav* OR integrated NEXT behav*):ab

#7 (health NEXT behav* OR “health knowledge” OR “health attitude*” OR health NEXT practice*):ab OR [mh “Health Behavior”] OR [mh “Attitude to Health”]
#8 (HbA1c OR A1c OR glyc* NEXT h?emoglobin OR glycoh?emoglobin OR glyc?emic NEXT control OR “glucose control” OR h?emoglobin NEXT a1c OR “mean glucose” OR “mean daily glucose” OR hypoglyc?emia OR hyperglyc?emia OR time NEXT in NEXT hypoglyc?emic NEXT range OR time NEXT in NEXT level NEXT 1 NEXT hypoglyc?emic NEXT range OR time NEXT in NEXT level NEXT 2 NEXT hypoglyc?emic NEXT range OR “time below range” OR TBR OR “time in target range” OR “time in range” OR TIR OR time NEXT in NEXT hyperglyc?emic NEXT range OR time NEXT in NEXT level NEXT 1 NEXT hyperglyc?emic NEXT range OR time NEXT in NEXT level NEXT 2 NEXT hyperglyc?emic NEXT range OR TAR OR “glucose variability” OR glyc?emic NEXT variability OR “estimated A1c” OR eA1C OR “glucose management indicator” OR GMI OR hypoglyc?emic NEXT episodes OR hyperglyc?emic NEXT episodes OR “area under the curve” OR AUC OR risk NEXT of NEXT hypoglyc?emia OR LBGI OR risk NEXT of NEXT hyperglyc?emia OR HBGI OR “time blocks” OR “quality of life” OR QoL OR “well being”):ab OR [mh “Glycated Hemoglobin A”] OR [mh “Quality of Life”] OR [mh “Glycemic Control”]
#9 (depress* OR anxiet* OR stress* OR distress* OR self-management OR self-care OR self-regulations OR self-monitoring OR adherence OR compliance OR cope OR coping OR resilien* OR self-efficacy OR behavioural control OR confidence OR self-esteem OR “family functioning” OR “family conflict” OR “family cohesion” OR “family flexibility” OR family NEXT relation* OR “family communication” OR “family problem solving” OR “family support” OR “family responsibility” OR “parental involvement” OR “family environment” OR “family adaptability” OR “social competence” OR “social skills” OR “social development” OR “social communication”):ab OR [mh Depression] OR [mh Anxiety] OR [mh “Stress, Psychological”] OR [mh “Psychological Distress”] OR [mh “Treatment Adherence and Compliance”] OR [mh “Self Efficacy”] OR [mh “Adaptation, Psychological”] OR [mh “Family Relations”] OR [mh “Social Skills”]
#10 #1 AND #2
#11 {OR #3-#7}
#12 #8 OR #9
#13 #10 AND #11 AND #12

OpenGrey (35 results)

Notes:

- OpenGrey Database is less sophisticated and so a simpler search was conducted searching for only the population of interest, returning few results.

Population Search: ("type 1 diabetes" OR “insulin-dependent diabetes”) AND (adolescent* OR youth* OR “young people” OR "emerging adult")
