# Supplementary material for: 24-Hour Movement Behaviours (Physical Activity, Sedentary Behaviour and Sleep) Association with Glycaemic Control and Psychosocial Outcomes in Adolescents with Type 1 Diabetes: A Systematic Review of Quantitative and Qualitative Studies
Source: Int J Environ Res Public Health. 2023 Feb 28;20(5):4363. doi: 10.3390/ijerph20054363 (PMC10001999; doi:10.3390/ijerph20054363)
Supplement: Supplementary file 1 [file ijerph-20-04363-s001.zip › Table S1 Inter-Rater Reliability Between Reviewers .pdf]

Table S1: Inter-Rater Reliability Between Reviewers

**Title and Abstract Inter-Rater Reliability**			
Reviewer 1	*Reviewer 2*	*Cohen's Kappa*	*Level of Agreement*
M.P.	B.H.	0.60	Moderate
M.P.	E.R.	0.72	Moderate
M.P.	S.M.	0.85	Strong
**Full Text Inter-Rater Reliability**			
Reviewer 1	*Reviewer 2*	*Cohen's Kappa*	*Level of Agreement*
M.P.	B.H.	0.69	Moderate
M.P.	E.R.	0.62	Moderate
M.P.	S.M.	0.64	Moderate
