# Supplementary material for: 24-Hour Movement Behaviours (Physical Activity, Sedentary Behaviour and Sleep) Association with Glycaemic Control and Psychosocial Outcomes in Adolescents with Type 1 Diabetes: A Systematic Review of Quantitative and Qualitative Studies
Source: Int J Environ Res Public Health. 2023 Feb 28;20(5):4363. doi: 10.3390/ijerph20054363 (PMC10001999; doi:10.3390/ijerph20054363)
Supplement: Supplementary file 1 [file ijerph-20-04363-s001.zip › Table S8 Characteristics of Qualitative Studies.pdf]

Table S8: Characteristics of Qualitative Studies

Author (Date), Location	Sample Characteristics *(N†, Age, Diabetes Duration, HbA1‡)*	Data Analysis Method	Movement Behaviour	Theme(s) and Related Outcome(s)	Quotation (s)
Bergner et al., (2018)<br>*USA, Nashville, Tennessee*	**N:** 25 adolescent-caregiver dyads<br>**Age (T1D Adolescent):** 15.56 ± 1.48 years<br>**Diabetes Duration:** 6.15 ± 4.04 years<br>**HbA1c:** 8.93 ± 1.95 %	Thematic Analysis	Sleep	**Theme:**<br>• Barriers to Obtaining Sufficient Sleep<br>**Primary:**<br>• Glycaemic Control<br><br>**Theme:**<br>• Diabetes Related Sleep Disruptions<br>**Secondary:**<br>• Self-Management	**Barriers to Obtaining Sufficient Sleep (Glycaemic Control):**<br>• "Usually [TEEN] sleeps fine, unless his blood sugar's high. Then he's up every couple hours" (Caregiver, p544).<br>• "it affects it [SLEEP] a little because like sometimes I have to wake up either to pee maybe because my blood sugar's high or I wake up feeling low and need to get something to eat" (Adolescent, p545).<br><br>**Diabetes Related Sleep Disruptions (Self-Management):**<br>• "You have to wake up more, because you have to check your sugar in the middle of the night if you need to" (Adolescent, p545).<br>• "I seem more lazy and it's where I don't want to test and I don't want to get up to eat, and if I do eat I don't test and I forget to take my insulin" (Adolescent, p546).<br>• "Generally if I don't get enough sleep… I'm not really on top of anything . . . I'm not my usual self. I don't like do things, I just kinda *sit*." (Adolescent, p546).<br>• "the lack of sleep would probably cause [her teen] to not be able to keep track of his blood sugars or test when he needs—it probably causes him to be a little lazy about it" (Caregiver, p546).
Quirk et al., (2014)<br>*UK, England*	**N:** 20 Parents<br>**Age (T1D Adolescent):** 10.8 ± 2.2 years	Thematic Analysis	Physical Activity	**Theme:**<br>• Parents Battle for Blood Glucose control<br>**Primary:**	**Parents Battle for Blood Glucose control (Glycaemic control):**<br>• "they do say when you exercise then you get better blood sugars, but I don't know, it just makes it more uncontrollable in some ways!" (Caregiver, p5).

	**Diabetes Duration:** 4.7 years ± 2.6 years<br>**HbA1c:** Not Stated			• Glycaemic control<br>**Secondary:**<br>• Coping	• “makes things easier to control” (Caregiver, p09), “the more sport he did that the less hypos he was having” (Caregiver, p19).<br>**Parents Battle for Blood Glucose control (Coping):**<br>• “a way of getting out his anger” (Caregiver, p6).
Rechenberg et al., (2018)<br>*USA, Connecticut*	**N:** 29 T1D Adolescents<br>**Age (T1D Adolescent):** 13.6 ± 1.9 years<br>**Diabetes Duration:** 5.6 ± 3.7 years<br>**HbA1c:** 8.3 + 1.3%	Thematic Analysis	Sleep	**Theme:**<br>• The never-ending cycle of disturbed sleep<br>**Secondary:**<br>• Anxiety	**The never-ending cycle of disturbed sleep (Anxiety):**<br>• “Because I’m scared that if I fall asleep and don’t respond to my receiver” (Adolescent, p550).<br>• “I panic a lot because one night my blood sugar dropped in the middle of the night and I ended up like having a seizure” (Adolescent, p550).<br>• “My mother sets an alarm for 2:30 in the morning and she comes and checks me so she doesn’t want me to have to get up in the middle of the night” (Adolescent, p550).
Ryninks et al., (2015)<br>*UK, England*	**N:** 12 T1D Adolescents<br>**Age (T1D Adolescent):** 14.5 ± 1.5 years<br>**Diabetes Duration: NS**<br>**HbA1c:** 8.3%.	Interpretive Phenomenological Analysis	Physical Activity	**Theme:**<br>• Benefits of Exercise<br>**Primary:**<br>• Glycaemic Control<br>• Quality of Life	**Benefits of Exercise (Glycaemic Control):**<br>• “keeps your blood sugar at good rates” (Adolescent, 13y, p4)<br>• ‘‘It helps to sort of control. I don’t really know why but I felt that um, if I’m doing more exercise um I can normally keep my levels at a more consistent rate” (Adolescent, 15y, p4).<br>• "Walking is really good for my blood sugars my mum says. I went on a walking weekend with Guides and my blood sugars were really good all the time I was doing that. So that’s good for my blood sugars" (Adolescent, 12y, p4).<br>• "Exercise kind of helps lower your sugar level. If it’s high then you just go outside for a run and then it goes back to normal again (Adolescent, 15y, p5).<br>**Benefits of Exercise (Quality of Life):**<br>• "When you’re doing exercise you know you’re helping your body as well as yourself" (Adolescent, 15y, p5).<br>• "I feel better for it but that’s probably just ‘cause I’m enjoying my sport" (Adolescent, 16y, p5).<br>• "It keeps you fit and um as well um if you um eat properly as well, then like I said it keeps you fit and nice and healthy" (Adolescent, 12y, p5).

Wenneick et al., (2009)<br>*Europe, Sweden*	**N:** 11 T1D Adolescents and Parents<br>**Age (T1D Adolescent):** 13 (11-16) years<br>**Diabetes Duration: NS**<br>**HbA1c:** Not Stated	Latent Content Analysis	Physical Activity	**Theme:**<br>• Physical activity as pleasure and annoyance<br>**Secondary:**<br>• Self-Management	**Physical activity as pleasure and annoyance (Self-Management):**<br>• "...Now I am going to start swimming four times a week... I have to do the homework some time before...it is all right but I have to monitor my blood sugar, and I have to take some extra insulin if it is too high, or some dextrose if it is too low. I might have to take dextrose before swimming, to make it a bit higher...and I have to go up and monitor my blood sugar somewhere in the middle of the swimming practice...." (Adolescent, p226).
Wilkie et al., (2017)<br>*UK, Scotland*	**N:** 16 T1D Adolescents<br>**Age (T1D Adolescent):** 11.6 ± 2.5 years<br>**Diabetes Duration:** 3.8 ± 4.3 years<br>**HbA1c:** Not Stated	Thematic Analysis	Physical Activity	**Theme:**<br>• Motivators Related to Health<br>**Primary:**<br>• Glycaemic Control<br>• Quality of Life<br><br>**Theme:**<br>• Family and Friends Participating<br>**Secondary:**<br>• Family Functioning	**Motivators Related to Health (Glycaemic Control):**<br>• '...when he has really high glucose late evening, we have two options: either extra jag or go for a walk and of course now he says "ok let's go for a walk"... after 15–20 minutes come back and sugar is fine' (Caregiver, p152).<br>**Motivators Related to Health (Quality of Life):**<br>• 'happy' or 'amazing' after exercise (Adolescent, p153).<br>**Family and Friends Participating (Family Functioning):**<br>• "because it's funner with other people, like you can keep motivated, but you can also have a laugh while you're doing it...,' (Adolescent, p153).
Quirk et al., (2016)<br>*UK, England*	**N:** 12 T1D Adolescents<br>**Age (T1D Adolescent):** 10.8 ± 0.9 years<br>**Diabetes Duration:** 4.3 ± 3.1 years	Thematic Analysis	Physical Activity	**Theme:**<br>• Children's physical activity is motivated by perceived positive outcomes, fun and enjoyment	**Children's physical activity is motivated by perceived positive outcomes, fun and enjoyment (Quality of Life):**<br>• "I feel quite satisfied" (Adolescent, p03), "cheerful" (Adolescent, p12) "I like walking because it really relaxes me" (Adolescent, p11).<br>**Children perceive difficulties that make physical activity harder (Glycaemic Control):**

	**HbA1c:** 55.1mmol/mol (Range = 41mmol/mol - 72mmol/mol).			**Primary:**<br>• Quality of Life<br>**Theme:**<br>• Children perceive difficulties that make physical activity harder<br>**Primary:**<br>• Glycaemic Control	• "Sometimes with football when my bloods go low and stuff ...I have to you know, come off or not go on or don't go on, just need to wait for them to come back up. [H: How does that make you feel?] Annoyed because not so long ago, there was a football match and I was supposed to be coming on at half- time, so I had to delay that as well, so I ended up not playing as long" (Adolescent, p03).
Blake et al., (2018)<br>*UK, England*	**N:** 11 Parents<br>**Age (T1D Adolescent):** 11.38 ± 2.69 years<br>**Diabetes Duration:** 6 ± 3.3 years<br>**HbA1c:** Not Stated	Thematic Analysis	Physical Activity	**Theme:**<br>• Benefits and challenges of PA with T1D<br>**Primary:**<br>• Glycaemic Control	**Benefits and challenges of PA with T1D (Glycaemic Control):**<br>• "'what we've learned is that physical activity keeps the spikes and the lows more moderate so you don't fluctuate as much... the physical activity just makes that more stable" (Caregiver, p4).

N, sample size; T1D, type 1 diabetes; HbA1c, glycated hemoglobin; PA, physical activity.
