# Supplementary material for: 24-Hour Movement Behaviours (Physical Activity, Sedentary Behaviour and Sleep) Association with Glycaemic Control and Psychosocial Outcomes in Adolescents with Type 1 Diabetes: A Systematic Review of Quantitative and Qualitative Studies
Source: Int J Environ Res Public Health. 2023 Feb 28;20(5):4363. doi: 10.3390/ijerph20054363 (PMC10001999; doi:10.3390/ijerph20054363)
Supplement: Supplementary file 1 [file ijerph-20-04363-s001.zip › Tables S4-S7 Movement Behaviours Associations with Primary and Secondary Outcomes.pdf]

Movement Behaviours Associations with Primary and Secondary Outcomes

Table S4: Physical Activity and Primary Outcomes

**Construct**	**HbA1c**			**QoL**			**CGM Metrics†**			**HbA1c** *n/N (%)*	**QoL** *n/N (%)*	**CGM Metrics†** *n/N (%)*
	F	*UF*	*NS*	*F*	*UF*	*NS*	*F*	*UF*	*NS*			
TPA	7	1	13	3	-	2	1	-	-	7/21*(33%)*	3/5*(60%)*	1/1*(0%)*
MVPA	7	1	12	6	-	1	-	-	1	7/20*(35%)*	6/7*(86%)*	0/1*(0%)*
VPA	3	-	5	-	-	-	-	-	-	2/8*(22%)*	-	-
MPA	2	-	5	1	-	-	-	-	-	2/7*(25%)*	1/1*(100%)*	-
LPA	-	-	4	1	-	-	-	-	-	0/4*(0%)*	1/1(100%)	-
**Total**	19	2	39	11	-	3	1	-	1	19/60*(32%)*	11/15*(79%)*	0/2*(0%)*

TPA, total physical activity; MVPA, moderate to vigorous activity; VPA, vigorous physical activity; MPA, moderate physical activity; LPA, light physical activity; HbA1c, haemoglobin A1c; QoL, quality of life; n, number of favourable associations, N, total number of associations; F, favourable, UF, unfavourable, NS, not significant

†Mean glucose metric utilised to determine associations

Table S5: Sedentary Behaviour and Primary Outcomes

**Construct**	**HbA1c**			**QoL**			**CGM Metrics†**			**HbA1c** *n/N (%)*	**QoL** *n/N (%)*	**CGM Metrics†** *n/N (%)*
	F	*UF*	*NS*	*F*	*UF*	*NS*	*F*	*UF*	*NS*			
Total SED	-	2	4	-	1	-	-	-	-	2/6*(33%)*	0/1*(0%)*	-
STC	-	4	3	-	-	-	-	-	-	4/7*(57%)*	-	-
STT	-	3	2	-	-	-	-	-	-	3/5*(60%)*	-	-
STS	3	-	-	-	-	-	-	-	-	0/3*(0%)*	-	-
TST	-	3	1	-	-	1	-	-	-	3/4*(75%)*	0/1*(0%)*	-
**Total**	3	12	10	-	1	1	-	-	-	12/25*(48%)*	0/2*(0%)*	-

SED, sedentary time; STC, screen time computer; STT, screen time TV; STS, screen time schoolwork; TST, total screen time; HbA1c, haemoglobin A1c; QoL, quality of life; n, number of unfavourable associations, N, total number of associations; F, favourable, UF, unfavourable, NS, no significance

†Mean glucose metric utilised to determine associations

Table S6: Sleep and Primary Outcomes

**Construct**	**HbA1c**			**QoL**			**CGM Metrics†**			**HbA1c** *n/N (%)*	**QoL** *n/N (%)*	**CGM Metrics†** *n/N (%)*
	F	*UF*	*NS*	*F*	*UF*	*NS*	*F*	*UF*	*NS*			
Duration	3	1	10	-	-	1	-	1	3	3/14*(21%)*	0/1*(0%)*	0/4*(0%)*
Continuity or Efficiency‡	1	-	4	-	-	-	1	-	2	1/5*(20%)*	-	1/3*(33%)*
Timing	-	2	1	-	-	-	-	-	-	0/3*(0%)*	-	-
Satisfaction/Quality	1	-	6	-	-	-	-	-	1	1/7*(14%)*	-	0/1*(0%)*
Alertness/Sleepiness	-	-	3	-	1	-	-	-	1	0/3*(0%)*	0/1*(0%)*	0/1*(0%)*
**Total**	5	3	24	-	1	1	1	1	7	5/32*(16%)*	0/2*(0%)*	1/9*(11%)*

HbA1c, haemoglobin A1c; QoL, quality of life; F, favourable, UF, unfavourable, NS, no significance; n, number of favourable associations, N, total number of associations

†Mean glucose metric utilised to determine associations

‡Where multiple measures of continuity and efficiency were used, the sleep efficiency construct was utilised to determine associations

Table S7: Movement Behaviour and Secondary Outcomes

**Movement Behaviour**	**Depression**				**Anxiety**				**Self-management†**			
	F	*UF*	*NS*	*n/N (%)*	*F*	*UF*	*NS*	*n/N (%)*	*F*	*UF*	*NS*	*n/N (%)*
**PA**												
TPA	-	-	-	-	-	1	1	0/2*(0%)*	1	-	-	1/1*(100%)*
MVPA	-	-	1	0/1*(0%)*	-	1	2	0/3*(0%)*	-	1	-	0/1*(0%)*
VPA	-	-	-	-	1	-	1	1/2*(50%)*	-	-	-	-
MPA	-	-	1	0/1*(0%)*	-	-	1	0/1*(0%)*	-	-	-	-
LPA	-	-	-	-	-	-	-	-	-	-	-	-
Total	-	-	2	0/2*(0%)*	1	2	5	2/8*(25%)*	1	1	-	1/ 2 *(50%)*
**SB**												
TSED	-	-	-	-	-	-	-	-	-	-	-	-
STC	-	-	-	-	-	-	-	-	-	-	-	-
STT	-	-	-	-	-	-	-	-	-	-	-	-
STS	-	-	-	-	-	-	-	-	-	-	-	-
TST	-	-	-	-	-	-	1	0/1*(0%)*	-	-	-	-
Total	-	-	-	-	-	-	1	0/1*(0%)*	-	-	-	-
**SLP**												

Duration	1	1	1	1/3*(33%)*	-	-	1	0/1*(0%)*	4	1	3	4/8*(50%)*
Continuity or Efficiency‡	1	-	-	1/1*(100%)*	-	-	-	-	-	-	4	0/4*(0%)*
Timing	-	-	-	-	-	-	-	-	-	1	-	0/1*(0%)*
Satisfaction/ Quality	3	-	-	3/3*(100%)*	-	-	-	-	1	1	3	1/5*(20%)*
Alertness/ Sleepiness	-	1	-	0/1*(0%)*	-	-	-	-	-	-	1	0/1*(0%)*
Total	5	2	1	5/8*(75%)*	-	-	1	0/1*(0%)*	5	3	11	5/19*(26%)*

Continued

**Movement Behaviour**	**Coping**				**Self-efficacy**				**Family Functioning**				**Social Competence**			
	F	*UF*	*NS*	*n/N (%)*	*F*	*UF*	*NS*	*n/N (%)*	*F*	*UF*	*NS*	*n/N (%)*	*F*	*UF*	*NS*	*n/N (%)*
**PA**																
TPA	1	-	-	1/1*(100%)*	-	-	-	-	-	-	-	-	-	-	-	-
MVPA	-	-	-	-	-	-	1	0/1*(0%)*	-	-	-	-	-	-	1	0/1*(0%)*
VPA	-	-	-	-	-	-	1	0/1*(0%)*	-	-	-	-	-	-	-	-
MPA	-	-	-	-	-	-	-	-	-	-	-	-	-	-	-	-
LPA	-	-	-	-	-	-	-	-	-	-	-	-	-	-	-	-
Total	-	-	-	-	-	-	2	0/2 *(0%)*	-	-	-	-	-	-	1	0/1 *(0%)*
**SB**																
TSED	-	-	-	-	-	-	-	-	-	-	-	-	-	-	-	-
STC	-	-	-	-	-	-	-	-	-	-	-	-	-	-	-	-
STT	-	-	-	-	-	-	-	-	-	-	-	-	-	-	-	-
STS	-	-	-	-	-	-	-	-	-	-	-	-	-	-	-	-
TST	-	-	-	-	-	-	-	-	-	-	-	-	-	-	-	-
Total	-	-	-	-	-	-	-	-	-	-	-	-	-	-	-	-
**SLP**																
Duration	-	-	1	0/1*(0%)*	-	-	-	-	-	-	-	-	-	-	-	-
Continuity or Efficiency‡	-	-	-	-	-	-	-	-	-	-	-	-	-	-	-	-
Timing	-	-	-	-	-	-	-	-	-	-	-	-	-	-	-	-
Satisfaction/ Quality	-	-	-	-	-	-	-	-	-	-	-	-	-	-	-	-
Alertness/	-	-	-	-	-	-	-	-	-	-	-	-	-	-	-	-

Sleepiness																
Total	-	-	1	0/1*(0%)*	-	-	-	-	-	-	-	-	-	-	-	-

PA, physical activity; TPA, total physical activity; MVPA, moderate to vigorous physical activity; VPA, vigorous physical activity; MPA, moderate physical activity; LPA, light physical activity; TSED, total sedentary behaviour; STC, screen time computer; STT, screen time television; STS, screen time schoolwork; TST, total screen time; F, favourable, UF, unfavourable, NS, no significance; n, number of favourable associations, N, total number of associations.
†Where multiple measures of self-management were reported (e.g., blood glucose checks and a measure of self-management) the measure of the construct was examined in relation to favourable/unfavourable associations due to it being a more complete measure of self-management.
‡ Where multiple measures of continuity and efficiency were used, the sleep efficiency construct was utilised to determine associations
